# Endogenous Hepatitis C Virus Homolog Fragments in European Rabbit and Hare Genomes Replicate in Cell Culture

**DOI:** 10.1371/journal.pone.0049820

**Published:** 2012-11-19

**Authors:** Eliane Silva, Sara Marques, Hugo Osório, Júlio Carvalheira, Gertrude Thompson

**Affiliations:** 1 Departement of Veterinary Clinics, Instituto de Ciências Biomédicas de Abel Salazar (ICBAS), Universidade do Porto, Porto, Portugal; 2 Centro de Investigação em Biodiversidade e Recursos Genéticos (CIBIO), Universidade do Porto, Vairão, Portugal; 3 Institute of Molecular Pathology and Immunology of the University of Porto (IPATIMUP), Porto, Portugal; University of Modena & Reggio Emilia, Italy

## Abstract

Endogenous retroviruses, non-retroviral RNA viruses and DNA viruses have been found in the mammalian genomes. The origin of Hepatitis C virus (HCV), the major cause of chronic hepatitis, liver cirrhosis, and hepatocellular carcinoma in humans, remains unclear since its discovery. Here we show that fragments homologous to HCV structural and non-structural (NS) proteins present in the European rabbit (*Oryctolagus cuniculus*) and hare (*Lepus europaeus*) genomes replicate in bovine cell cultures. The HCV genomic homolog fragments were demonstrated by RT-PCR, PCR, mass spectrometry, and replication in bovine cell cultures by immunofluorescence assay (IFA) and immunogold electron microscopy (IEM) using specific MAbs for HCV NS3, NS4A, and NS5 proteins. These findings may lead to novel research approaches on the HCV origin, genesis, evolution and diversity.

## Introduction

Viruses may represent genetic elements that gained the ability to move between cells, or represent previously free-living organisms that became parasites, or they may be the precursors of life [1]. The only group of viruses known to have left a fossil record in the form of endogenous provirus’s are the retroviruses. There are studies describing that 8% of the human genome is made up of these elements [2], [3]. Many other viruses, like non-retroviral RNA viruses, are known to generate DNA forms of their own genomes during replication [4]. Recently a handful of endogenous retroviruses, non-retroviral RNA viruses and DNA viruses have been found in mammalian genomes. Among these, Human Retrovirus 5 and endogenous lentivirus were found to be present in European rabbits [5], [6], and endogenous bornaviruses, filoviruses and parvoviruses have been described in mammalian genomes [7], [8]. Calicivirus associated with hemorrhagic diseases in European rabbits and hares (*Lepus europaeus* and *Lepus timidus*) have also been described [9].

The origin of the Hepatitis C virus (HCV) remains unclear since its discovery, largely because a closely related animal homolog virus was not identified, until a recently description of a canine homolog of HCV [10]. Unlike most RNA viruses which usually cause acute diseases, HCV establishes life-long, persistent, intrahepatic infections in the majority of the infected individuals, frequently leading to the development of cirrhosis and hepatocellular carcinoma and affects about 170 million people worldwide [11], [12]. HCV is a positive single-stranded RNA virus, so called because upon infection their RNA can be directly translated into protein by the host machinery, with an envelope that belongs to the *Flaviviridæ* family [11]. Its genome, of ∼9.6 kb, contains one large open reading frame that encodes one large polyprotein that is processed by viral and cellular proteinases to produce the virion structural proteins (core and envelope glycoprotein’s E1 and E2) as well as non-structural (NS) proteins (P7-NS2-NS3-NS4A-NS4B-NS5A-NS5B) that form the replication complex [11]. NS3 to NS5B proteins are necessary and sufficient to establish membrane-bound replication complexes that catalyze RNA replication and NS5B protein codes RNA-dependent RNA polymerase (RdRp), because it has a Gly-Asp-Asp (GDD) motif which serves to replicate the HCV-RNA genome [11], [13], [14].

Chronic HCV infection is currently treated with a combination of pegylated interferon-α and ribavirin but is not always efficient [15]. There are six main genetic types of hepatitis C virus and more than 80 subtypes [16]. The development of antiviral drugs and the molecular studies of HCV have been hampered by a lack of a reliable cell culture system like hepatoma cell lines, African green monkey Vero cells, mosquito cells allowing a persistent *in vitro* virus replication and viral adaptation to the culture [17]–[20]. To evaluate virus replication in cell cultures, immunofluorescence assay (IFA) and immunogold electron microscopy (IEM) methods among others, were described in previous studies [21], [22].

The European rabbit, native to the Iberian Peninsula, is the single recognized progenitor of domestic rabbits [23]. Rabbits have many hereditary diseases common to humans like aortic arteriosclerosis, hypertension, hypertrophic cardiomyopathy, osteoporosis, making them a valuable model in both biomedical and fundamental research [23].

During an investigation addressing the *Flaviviridae* family in the search for significant natural virus’s reservoirs of animal diseases we have tested body fluids and liver homogenates samples from domestic, wild rabbits and hare for Bovine Virus Diarrhoea Virus (BVDV) antigen detection using a commercial blocking ELISA (SerelisaTM BVD p80 Ag Mono Indirect, Symbiotics, Lyon - France) and all tested positive. However, when we tested serum and body fluid samples of those animals in the study using an antibody ELISA (SERELISA® BVD p80 Ab Mono Blocking detection kit) that detects specific antibodies to a protein common to all strains of bovine viral diarrhea/mucosal disease (BVD/MD) and border disease (BD) virus (p80/125 non-structural protein) they all tested negative. Efforts to detect significant *Pestivirus* proteins were unsuccessful. These observations and other findings (not shown), led to hypothesize that potential protein cross-reactivity within members of the *Flaviviridae* family could explain the results in that preliminary work.

In this study we were able to detect that homologous DNA fragments coding for HCV core, envelope glycoprotein’s E1 and E2, protease NS2–3, serine protease NS3, NS4A, NS5A and the NS5B specific proteins were endogenous in the European rabbit and *Lepus europaeus* genomes. To test this hypothesis, genomic fragments, of structural and NS proteins were tested by RT-PCR, PCR, one dimension gel electrophoresis (1-DE), MALDI-TOF/TOF mass spectrometry including MS/MS peptide sequencing. Blastn with HCV 1b D90208 (HCV database), *Oryctolagus cuniculus* (*O. cuniculus*) and *Homo sapiens* (*H. sapiens*) genomes in Ensembl website (www.ensembl.org) was conducted to evaluate homologies between the virus and the referred species. Moreover, blastn in the same website between *O. cuniculus* and primers used in this study was also performed. To validate the unexpected findings, and to test the biological significance of the HCV homologue detected fragments, suspensions of liver homogenates in this study were inoculated in Mardin-Darby Bovine Kidney (MDBK) cell line and bovine testis (BT) primary cell cultures. HCV fragment homologues replication was detected by IFA and IEM with MAbs for NS3, NS4A and NS5 HCV specific proteins.

## Results

### RT-PCR, PCR and Sequencing of Rabbit and Hare

To investigate if HCV genomic fragments are present in the European rabbit and *Lepus europaeus* genomes we performed RT-PCR and PCR using extracted RNA and DNA respectively from liver homogenates and sequenced all amplified products obtained with these methods. Core, E1/E2, NS5A/B and NS5B HCV genomic proteins were successfully amplified from all samples in this study (Figure S1. A–H), when specific primers were used in both PCRs as previously described [24]–[28]. RT-PCR from NS5A/B HCV proteins was also performed in WBC and serum samples from 5 domestic rabbits and in body fluids from 6 wild rabbits and one hare (Figure S1. I–J). Amplified fragments were detected only from WBC. Sequencing of RT-PCR and PCR amplified regions was performed.

Alignments and tblastn searches were conducted as a query in the HCV database and whole-genome shotgun in rabbit genome resources at GenBank, NCBI. Retrieved fragment sequences from core, E1/E2, NS5A/B and NS5B HCV specific proteins with 80 to 100% of nucleotides (nt) identities and blast E-values of 8E-09 to 2.4 were identified (Table 1). HCV blast results of selected sequences and regions can be seen in the supporting information, presenting all blasted identities and genotype variability for the core (Table S1), E1/E2 (Table S2) and NS5B (Table S3) proteins.

**Table 1:** Endogenous HCV fragment sequences generated by PCR and RT-PCR from the European rabbit *and Lepus europaeus* liver samples.

HCVregion	Primers	Identified nucleotides	Acessionno.	Genotype	Position	E-value	% Identities
Core	104/134	**GTGACCGCTCGGAAGTCTTCC** a	GU441256	1b	304-284*	0.005	100
Core	104	**CGAGGTTCCCGTCCCTCTTGG**	U10230	2a	301–321	0.33	100
Core	134	**CGACCGCTCGGAAGTCTTCCTA**	HM049503	2a	508-487	0.004	100
E1/E2	HVR1F/R	**TTTGGAAAAGGCCAG** G**GAA**	AM885177	1a	232-214*	0.37	94
E1/E2	HVR1R	**TT**T**TGTT**TC**CGAAG**C**CAATACATCCA** b	AY767496	3a	133–158	0.12	84
E1	HVR1F	**CC** A**C**C**AA**A**AC**CG**TC**C**CTGGCCTTTTCCAAA**	AM885166	1a	203–232	2.4	80
NS5B	AS/S	**GC** T**GG**G**G**A**CTTGTGCCTTCAGG**GGA**CATGTGG**	EF116149	6a	235-204*	0.014	81
NS5B	S	**AGCCTCCGTGAAAGCTTG **	HM009084	1b	303-286*	1.1	94
NS5B	S	**CAAGCTTTCACGGAGGCTATGACCAGGAATTCCGCC**	AY685047	4p	366–401*	4e-08	100
NS5B	AS	**AATCAAAGCAGCGGGTATCATACGGGATCCCCA**	AY743071	4p	33-1*	8e-07	100
NS5B	S`	**TCTTCACGGAGGCTATGACTAGGTATTCTGCC**	GU589872	1	348–379	2e-04	93
NS5B	AS`	**GATCCCGTATGATACCCGCTGCTTTGA**	DQ508484	1b	5–31	6e–06	100
NS5B	AS`	**TCAAAGCAGCGGGTATCATACGGGATCCCCA**	DQ345619	1b	32-2*	6e-05	100
NS5B	Pr3/Pr4	**GC** T**GG**G**G**A**CTTGTGCCTTCAGG**GGA**CATGTGG**	EF116149	6a	235-204*	0.014	81
NS5B	Pr3/4	**AGCTTTCACGGAGGCTATGACCAGGTACTCAGC** c	DQ238690	2b	66–98	8e-09	100
NS5B	Pr3/4	**AGCTTTCACGGAGGCTATGACCAGATATTCAGCA**	DQ663603	3a	327–360	4e-07	100
NS5B	Pr4	**TATGATACCCGCTTGCTTTGA**	X88622	1a	14–29	1.1	100
NS5B	Pr4	**GTAGAGTCGAAGCAACGGGTA** A**CATA**	EU255955	1a	8190-8165	0.023	96

Nucleotide homology between the studied samples and HCV sequences deposited at the site (http://hcv.lanl.gov/content/sequence/BASIC_BLAST/basic_blast.html) are labeled in bold and underlined. See Supplementary Information for whole blast,

a Table S1.,

b Table S2,

c Table S3.

* Reverse complement.

When blastn of HCV 1b D90208 (HCV database) complete sequence to *O. cuniculus* and to *H. sapiens* genomes was performed in the Ensembl database, match of HCV homolog fragments were detected only for *O. cuniculus* (Table S4). However, blastn against all independent HCV regions to both species showed match of virus homolog fragments for all HCV regions except NS2 and NS5B for *O. cuniculus* and only the HCV 3` untranslated region for *H. sapiens* (Table S4). When blastn of all PCR primers used in this study were performed in Ensembl website, they showed a match with *O. cuniculus* except for HVR1F, Pr3 and Pr4 from E1/E2 and RdRp-NS5B HCV specific regions respectively, as they are degenerated primers (Table S6). Detected homology for both blastn performed showed high percentage of nt identities but was not statistically validated as high E-values were obtained. However, virtually all identical short alignments have relatively high E-values because the calculation of the E-values takes into account the length of the query sequence and shorter sequences have a higher probability of occurring in the database purely by chance [29].

These results suggest some genetic correlations between HCV and *O.cuniculus* and that the matched small HCV homolog fragments are present as repeated sequences throughout *O. cuniculus* genome as identified in PCRs and mass spectrometry results.

### Analysis of Rabbit and Hare by MALDI-TOF/TOF

Liver homogenate proteins were separated by SDS-PAGE and stained with Coomassie Blue. The visible protein bands that were selected on the basis of their molecular weight and that could harbor proteins matching the core, envelope glycoprotein’s E1 and E2, serine protease NS3, NS4B, NS5A and NS5B HCV genomic proteins, were excised from the gel, and analyzed by MALDI-TOF/TOF-MS/MS as described by Pinho *et al*. [30]. For each digested sample, the MS and MS/MS spectra were combined for the MASCOT database search. With the matched MS/MS spectra of the European rabbit and *Lepus europaeus* to HCV sequences deposited at Swiss-Prot/UniProt protein database a total of 34 peptide sequences were identified, such as core, envelope glycoprotein’s E1 and E2, protease NS2–3, serine protease NS3, NS5A and RdRp-NS5B HCV specific proteins (Table 2). Some MS/MS spectra were retrieved with confidence interval (C.I.)<95%, however they were validated by the associated detected match mass error (−0.08 to 0.09 Da). Among these, for domestic rabbit we were able to identify 16 peptide sequences that match core, envelope glycoprotein’s E1 and E2, protease NS2–3, serine protease NS3, NS5A and RdRp-NS5B HCV specific proteins with a C.I. of 71 to 99%, E-value 0.013 to 1.1 and match mass error of −0.08 to 0.09 Da (Table 2, S5). In wild rabbits, 12 peptide sequences that match to the core, serine protease NS3, NS5A and RdRp-NS5B HCV specific proteins were identified with a C.I. of 22 to 93%, E-value 0.016 to 1.1 and match mass error of −0.08 to 0.06 Da (Table 2, S5). Lastly, from hare we were able to identify 6 peptide sequences that match core, envelope glycoprotein E2, protease NS2–3, NS5A and RdRp-NS5B HCV specific proteins with a C.I. of 53 to 82%, E-value 0.067 to 0.92 and match mass error of −0.07 to 0.04 Da (Table 2, S5). Rabbit liver samples were fresher than the corresponding from the hare sample, which could explain the less number of sequence identities detected in the latter specie. Spectra generated by MS/MS analysis from all identified peptide sequences can be observed in Figure S2. NS4B protein could not be identified by MS/MS analysis in our study.

**Table 2:** HCV homolog proteins identified by MALDI-TOF/TOF-MS/MS analysis from the European rabbit and *Lepus europaeus* liver homogenates.

Animal	Accession no.†(genotype)	Proteinname	Best peptidesequence	Genomeposition (aa)	Matcherror (Da)	Total ionscore C I %	E-value
DR	POLG_HCVJA (1b)	Core	STNPKPQR	2–9	−0.02	99	0.39
DR	POLG_HCVJA (1b)	Core	SQPRGRR	56–62	−0.02	99	0.23
DR	POLG_HCVJA (1b)	Core	TWAQPGYPWPLYGNEGMGWAGWLLSPR	75–101	0.07	99	0.3
DR	POLG_HCVJA (1b)	E1	NSSIPTTTIRR	250–260	−0.04	99	0.022
DR	POLG_HCVJA (1b)	E2	VASSTQSLVSWLSQGPSQK	392–410	−0.07	98	0.046
DR	POLG_HCVVA (2K)	E2	SIEEFR	461–466	0.03	71	0.58
DR	POLG_HCVBK (1b)	NS2–3	KVAGGHYVQMAFMK**	927–940	−0.04	95	0.047
DR	POLG_HCVJA (1b)	NS3	GPITQMYTNVDQDLVGWPAPPGAR	1095–1118	0.06	81	0.6
DR	POLG_HCVJ1 (1b)	NS3	AVDFIPVESLETTMR	1192–1206	−0.07	87	0.049
DR	POLG_HCVJA (1b)	NS5A	DVWDWICTVLSDFKTWLQSKLLPR*	1979–2002	0.09	81	0.013
DR	POLG_HCVT5 (6b)	NS5A	IPGIPFISCQAGYR*	2008–2021	−0.01	89	0.15
DR	POLG_HCVK3 (3a)	NS5A	NGSMRLAGPR**	2047–2056	−0.02	74	0.92
DR	POLG_HCVSA (5a)	NS5A	GSPPSLASSSASQLSAPSLK	2194–2213	−0.08	90	0.087
DR	POLG_HCVJA (1b)	NS5B	VEFLVNTWK	2620–2628	0.01	81	0.15
DR	POLG_HCVSA (5a)	NS5B	AAIRSLTQR	2674–2682	−0.02	99	0.1
DR	POLG_HCVJA (1b)	NS5B	AFTEAMTR	2757–2764	−0.01	81	1.1
WR	POLG_HCVNZ (3a)	Core	SQPRGRR	56–62	0.04	22	1.1
WR	POLG_HCV6A (6a)	NS3	CDELAGKLKSLGLNAVAFYR*	1405–1424	−0,04	87	0.15
WR	POLG_HCVJ8 (2b)	NS3	GRLGVYR	1498–1504	−0.04	57	0.05
WR	POLG_HCVJF (2a)	NS3	AKAPPPSWDAMWKCLAR*	1601–1617	0.04	93	0.016
WR	POLG_HCVVO (6K)	NS5A	NGSMRISGSR	2043–2052	0.04	22	0.05
WR	POLG_HCVVN (6d)	NS5A	IVGPKMCSNVWNNR*	2044–2057	0.01	87	0.2
WR	POLG_HCVCO (1b)	NS5A	VGDFHYVTGMTTDNVK**	2096–2111	0.06	74	0.5
WR	POLG_HCVCO (1b)	NS5A	GSPPSLASSSASQLSAPSLK	2193–2212	−0.08	74	0.53
WR	POLG_HCVJ6 (2a)	NS5A	SDLEPSIPSEYMLPKKR	2264–2280	−0.05	22	0.1
WR	POLG_HCV6A (6a)	NS5B	SASLRQK	2472–2478	0.00	87	0.13
WR	POLG_HCVJ8 (2b)	NS5B	LLTVEEACALTPPHSAK*	2524–2540	−0,05	57	0.15
WR	POLG_HCV6A (6a)	NS5B	MALYDVTR**	2601–2608	0.01	87	0.2
Hare	POLG_HCVJP (2b)	Core	GSRPTWGPSDPRHR	102–115	0.04	53	0.13
Hare	POLG_HCVJP (2b)	E2	LWHYPCTVNFTIFKVR*	619–634	−0.04	53	0.65
Hare	POLG_HCVJK (3K)	NS2–3	LGKEVLLGPADDYR	1011–1024	−0.07	81	0.067
Hare	POLG_HCVK3 (3a)	NS5A	NGSMRLAGPR**	2047–2056	−0.05	67	0.92
Hare	POLG_HCVJP (2b)	NS5B	AASKVSAR	2516–2523	0.00	53	0.65
Hare	POLG_HCVT5 (6b)	NS5B	DVRSHTSK	2535–2542	0.04	82	0.09

DR – Domestic rabbit *(Oryctolagus cuniculus)*, WR – Wild rabbit *(Oryctolagus cuniculus.)*, Hare (*Lepus europaeus*),

† – SwissProt accession number,

* Modification – Carbamidomethyl (C),

** Modification – Oxidation (M).

The most representative HCV genotype that matches with the identified peptide sequences is 1b. Furthermore, when we matched all identified peptide sequences with the Swiss-Prot accession number POLG_HCVJA (genotype 1b), a total genome polyprotein coverage of 12% (351/3010 aa) (Figure S3) was obtained. It is important to note that these match cover 28% (53/190 aa) of the core, 6% (11/192 aa) of the E1, 9% (34/363 aa) of the E2, 22% (47/217 aa) of the NS2–3, 12% (73/631aa) of the NS3, 17% (74/447 aa) of the NS5A and 10% (59/591 aa) of the RdRp-NS5B structural and NS HCV 1b genome polyprotein.

### IFA and IEM of Rabbit and Hare in Bovine Cell Cultures

**Figure 1:**
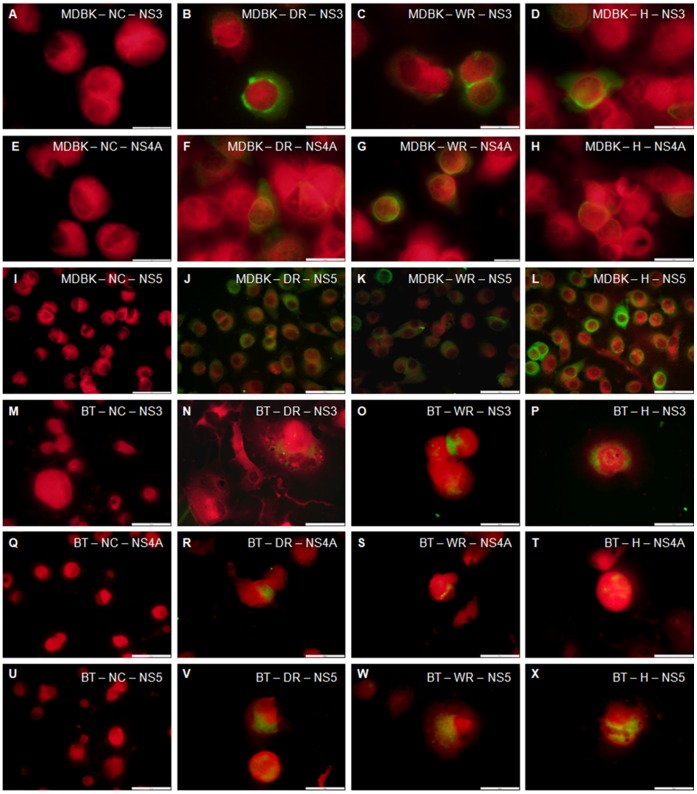
Detection of HCV proteins by immunofluorescence in MDBK and BT cells. The cells infected with liver homogenates from domestic (DR), wild (WR) rabbits and a hare (H) were analyzed 14 days post infection. MDBK cells incubated with MAbs specific for NS3 (A to D), NS4A (E to H) and NS5 (I to L) proteins. BT cells incubated with MAbs specific for NS3 (M to P), NS4A (Q to T) and NS5 (U to X) proteins. Negative controls (NC) for each cell culture and each Mab was also performed (A, E, I, M, Q and U). No fluorescence was detected. Scale bars: (A to H), 20 µm; (I to X), 50 µm.

**Figure 2:**
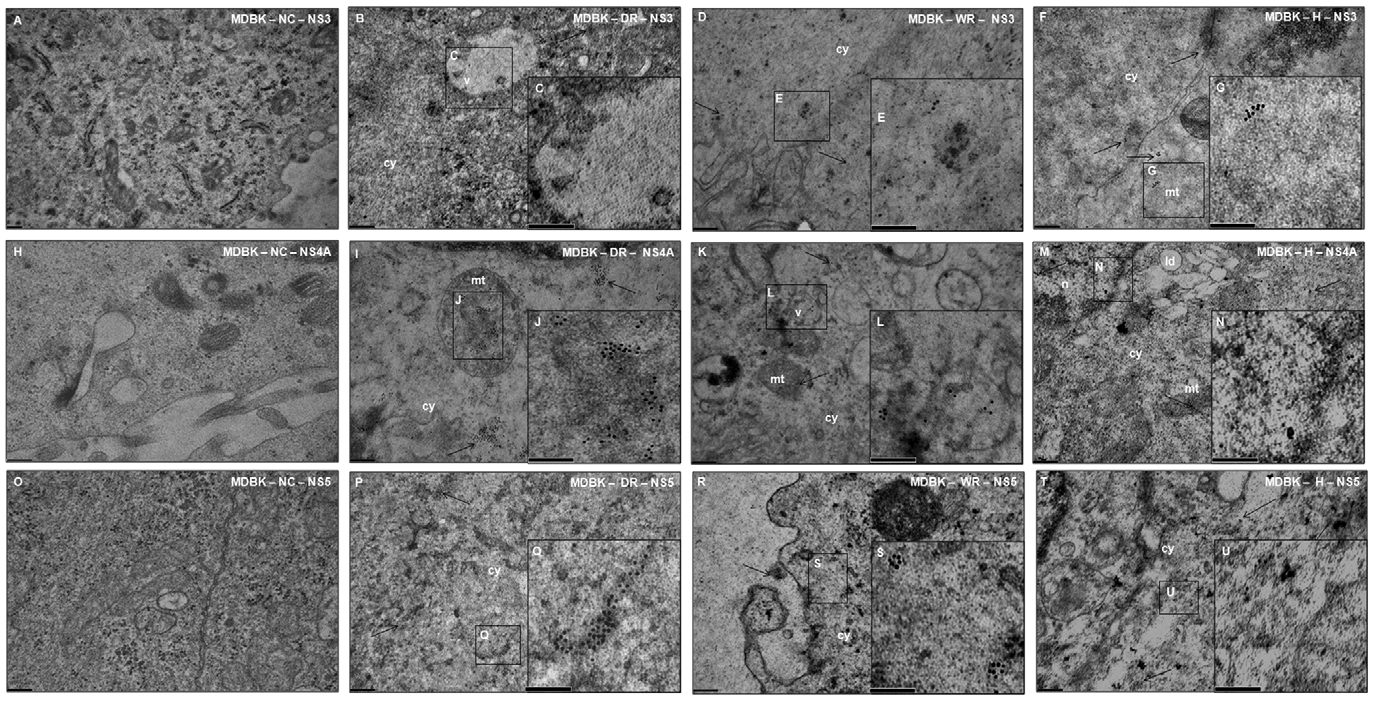
Immunogold TEM detection of HCV proteins in MDBK cells. The cells infected with liver homogenates from domestic (DR), wild (WR) rabbits and a hare (H), were analyzed 14 days post infection. The cells were incubated with MAbs specific for NS3 (A to F), NS4A (H to M) and NS5 (O to T) proteins. High-magnification views of immunogold particles in several organelles are shown in boxes (C, E, G, J, L, N, Q, S and U). The immunogold particles were detected in vacuoles (v) (C and L), in mitochondria (mt) (G and J), cytoplasm (cy) (E, Q, S and U) and nucleous (n) (N). Arrows indicate the presence of gold particles detected in the same organelles (mt, cy) and close to the extracellular membrane (R). Negative controls (NC) for each MAb were also performed (A, H and O). No immunogold particles were detected. The immunogold particles were 10 nm in diameter. Scale bars: (A) 0.2 µm; (B, D, F, H, I, K, M, O, P, R and T), 200 nm; (C, E, G, J, L, N, Q, S and U), 100 nm.

To address the biological significance of these findings and of the question whether these HCV homolog fragments can replicate in cell culture, MDBK cell line and primary BT cell cultures were inoculated with suspensions of liver homogenate samples from the wild, domestic rabbits and the hare. Inoculated cells were harvested at the second subculture (14 days post infection) and tested by IFA using mouse MAbs anti-HCV NS3, anti-HCV NS4A and anti-HCV NS5, specific immunostaining for the three selected antibodies could be demonstrated (Figure 1). As MDBK cell line showed a better signal for European rabbit and *Lepus europaeus* endogenous HCV homolog fragments replication than BT primary cell cultures by IFA, they were selected for treatment by IEM (Figure 2). Negative controls (uninoculated MDBK and BT cells) were included and treated with the selected MAbs and no reaction was detected (Figure 1, 2).

## Discussion

Hepatitis C virus, whose origin remains unclear, is the major cause of chronic hepatitis, liver cirrhosis and hepatocellular carcinoma in humans [11]. The HCV diagnosis in humans is usually done using serum or plasma of infected patients to detect virus or specific antibodies in circulation [31]. The absence of amplicons from serum and body fluids in the present study is an indication that viral particles were not in circulation. On the other hand, all positive samples were originated from samples containing only cells, suggesting that the detected virus fragments are endogenous in the European rabbit and *Lepus europaeus* genomes.

Endogenous *flaviviruses* have previously been reported in the genomes of *A. aegyptii* and *A. albopictus* mosquitoes [32], [33] and complete putative genomic structures have not been determined. Moreover, recently *Flavivirus* were also detected in mosquito species using degenerated primers designed for a *Flavivirus* generic RT-nested-PCR assay [34], [35] what is in agreement with our results, when testing rabbits and hare samples by PCR and RT-PCR using HCV specific primers. Endogenous viral elements have also been identified and demonstrated in animal genomes, and it has been suggested that its diverse sequences may account for the gene flow [36] between animals and viruses.

The sequenced fragments in our study were homologous to various HCV types, and included regions that are commonly used in HCV research or diagnosis (Table 1). The detected fragments belong to conserved regions in the virus, which are common to several types and/or subtypes (Table 1, S1, S2, S3). These findings demonstrate that these fragments are endogenous in the European rabbit and *Lepus europaeus* genomes with a high probability to appear as repeated sequences throughout their genome as identified when HCV 1b D90208 and HCV specific primers blastn were performed in the Ensembl website against the *O. cuniculus* genome (Table S4, S6). The findings could also suggest that the different animals could have equal and different HCV homolog specific peptides in their genomes (Table 2) and therefore, excluding the possibility of contamination between samples. All the identified peptides are located in HCV specific proteins which have several important determinants or functions like for example host/cell tropism, viral immunogenicity and participation in virus replication complex. Furthermore, the obtained results demonstrate that the studied species have some homologous genetic make-up with the members of the *Flaviviridae* family. Miller and Purcell [37] described significant homology between HCV and two *Pestivirus* polyproteins, which is in line with our results.

The analysis of the sequences that were generated either by PCR or MALDI techniques showed a considerable degree of diversity, which is similar to what is observed for HCV in nature. This diversity explains, at least in part, the difficulties involved in the development of effective vaccines and therapies for the disease [38], [39]. However, more studies should be performed to evaluate the significance of the peptide sequences distributed along the HCV genome and which are endogenous to the genomes of the studied species.

We demonstrate that HCV homologous fragments present in the rabbit and hare genomes can replicate in bovine cell cultures (Figure 1, 2), although no virus-like particles were detected in the liver homogenates of the tested samples. In the cell negative controls no immunofluorescence or immunogold particles were detected. Immunogold particles were detected in different cell organelles such as vacuoles, nucleus, lipid droplets, mitochondria and cytoplasm consistent with others reports previously described for HCV replication in cell cultures [40], [41]. These findings may be an insight into new approaches for therapy and molecular studies.

This study provides the evidence of endogenization of HCV homolog fragments in European rabbit and *Lepus europaeus* genomes and demonstrates that the detected fragments are infectious and replicate in bovine cell culture systems. Our findings may lead to novel research approaches on the HCV origin, genesis and diversity. The timeframe of the HCV integration event in the rabbit genome or virus origin are still to be determined and warrants further molecular, biologic and phylogenetic investigations. The biological and epidemiological implications of these findings, which have to be further explored, may also suggest the European rabbit and *Lepus europaeus* as possible candidate models for functional and evolutionary studies.

## Materials and Methods

### Ethics Statement

Animal Care and Use Committee approval was not obtained for this study because bovine testicles were collected from bovine fetuses after the dam’s slaughter at an authorized slaughterhouse. Animals were protected at slaughter according to the Council Directive 93/119/EC of 22 December 1993, and bovine testicles collection for experimental purposes was done by qualified members as previously authorized by the Portuguese National Authority for Animal Health (Direcção Geral de Veterinária) of the Ministry for Agriculture, Sea, Environment and Spatial Planning (Ministério da Agricultura, do Mar, do Ambiente e do Ordenamento do Território).

### Samples

Liver homogenates from 6 wild and 6 domestic rabbits and 1 hare, 5 serum and WBC samples of domestic rabbits and 7 samples of body fluids of the 6 wild rabbits and the hare were used in this study. These samples belong to a collection of the Laboratory of Infectious Diseases of the Veterinary Clinics Department at ICBAS, University of Porto [42], [43].

### RNA, DNA Extractions and cDNA Synthesis

Total RNA was extracted from 10% (w/v) liver homogenates with TRI® Reagent LS (Sigma-Aldrich, Steinheim, Germany). From the RNA extracts cDNA was synthesized using random hexamers (Amersham, New Jersey, USA). Briefly, 5 µl of RNA was mixed with 2 µl of diethyl pyrocarbonate (Sigma-Aldrich, Steinheim, Germany) water and 1 µl (0.02 U) of random hexamer, denaturated at 65°C for 10 min and then cooled on ice for 10 min. Thereafter, the hexamers were extended for 90 min at 37°C by 1 µl Moloney murine leukaemiavirus reverse transcriptase (200 U) (Invitrogen™, Paisley, UK) [44]. in the presence of 2.0 µl dNTPs (10 mM), 5 µl 1^st^ standard buffer and 1 µl RNAse inhibitor (Roche, Mannheim, Germany). The enzyme was then inactivated for 5 min at 95°C and the cDNA was immediately used or kept at −20°C until further processed. Genomic DNA was extracted from the same samples as RNA using the QIAamp® DNA blood kit (Qiagen, Hilden, Germany) according to the manufacturer instructions.

### RT-PCR, PCR and Sequencing of the Amplified Fragments

Using cDNA and DNA, RT-PCR and PCR were performed targeting the specific HCV structural and NS proteins, core, envelope glycoprotein’s E1/E2, NS5A/B and NS5B [24]–[28], in a thermocycler C 1,000 (Bio-Rad, California, USA). Briefly, the PCR mix (25 µl) contained 2.5 µl of 10× PCR buffer, 0.6 µl dNTPs (10 mM), 10 pmol of each primer, 2.5 µl MgCl_2_ (25 mM) and 1 U Taq DNA polymerase (Fermentas, St. Leon-Rot, Germany). PCR cycling conditions were performed as described above with the following primers: sense no. 256 (5′ CGCGCGACTAGGAAGACTTC 3′) and antisense no. 186 (5′ ATGTACCCCATGAGGTCGGC 3′) for the core PCRI; sense no. 104 (5′ AGGAAGACTTCCGAGCGGTC 3′) and antisense no. 134 (5′ CCAAGAGGGACGGGAACCTC 3′) for the core PCRII; sense HVR1F (5′ TGCTGGGTCCARRTYACCCC 3′) and antisense HVR1R (5′ GCTGTCATTACAGTTAAGGGCA 3′) for the E1/E2 (HVR-1); sense S (5′ TGGGGATCCCGTATGATACCCGCTGCTTTGA 3′) and antisense AS (5′ GGCGGAATTCCTGGTCATAGCCTCCGTGAA 3′) for the NS5A/B PCRI; sense S′ (5′ TGCGGTTATTGCCGTTGTCGCGCCAGCGG 3′) and antisense AS′ (5′ GGCAGAATACCTAGTCATGGCCTCTGTGAA 3′) for the NS5A/B PCRII; sense Pr3 (5′ TATGAYACCCGCTGYTTTGACTC 3′) and antisense Pr4 (5′ GCNGARTAYCTVGTCATAGCCTC 3′) for the RdRp-NS5B. The amplified products were analyzed on a 1.2% (w/v) agarose gel stained with gel red™ (Biotium, California, USA) and visualized under UV light (Bio-Rad, California, USA). The amplicons were directly sequenced at STAB Vida (C. Caparica, Portugal) after being purified using a Qiagen kit (Qiagen, QIAquick® Gel Extraction Kit, Crawley, UK) using the same primers.

### Proteins Extraction and Quantification

Proteins were extracted from 10% (w/v) liver homogenates following the Alliance for Cellular Signaling (AFCS) protocol [45]. Protein concentration was determined in a Qubit® Fluorometer (Invitrogen, Carlsbad, USA) according to the manufacturer instructions.

### Proteomic Analysis by 1-DE and MALDI-TOF/TOF-MS/MS

SDS-PAGE, 1-DE, was performed by Laemmli method [46] After extraction, proteins were separated by 12% SDS-PAGE using 30 µg of total protein in a Mini-Protean® 3 Cell (Bio-Rad, California, USA) system. After SDS-PAGE separation, proteins were Coomassie blue stained (Imperial Protein stain, Thermo Scientific, Rockford, USA).The protein bands of interest were reduced, alkylated and *in gel* digested with trypsin accordingly to trypsin manufacturer’s suggested protocol (Promega, USA). The resulting peptides were concentrated with ZipTips (Millipore, USA) in agreement with manufacturer’s instructions and eluted into the MALDI plate using alpha-cyano 4-hydroxycinnamic acid as a matrix as already described [30]. MS and MS/MS peptide mass spectra were acquired with a MALDI-TOF/TOF 4700 Proteomics Analyzer (ABSCIEX, USA). Peptide mass spectra were obtained in reflector positive mode for a mass window of 700–4000 Da. Some peptides were selected for MS/MS fragmentation by collision induced dissociation (CID) in MS/MS positive mode. Proteins were identified using the combined information of MS and MS/MS spectra by the GPS (Global Proteome Server) Explorer Software v3.6 (ABSCIEX, USA) which integrates the Mascot protein search engine v2.1.04 (Matrix Science, UK) configured to perform searches at the Swiss-Prot/UnitProt database. This procedure was performed at the Institute of Molecular Pathology and Immunology of the University of Porto (IPATIMUP) Proteomics Unit.

### Cell Culture and Sample Inoculation

MDBK cells (American Type Culture Collection) [47] obtained from the Friedrich-Loeffler-Institute (Insel Riems, Germany) were maintained at 37°C, 5% CO_2_ in D-MEM (Invitrogen™, Paisley, UK) supplemented with 10% heat-inactivated horse serum (Invitrogen™, Paisley, UK), penicillin (100 U/ml) – streptomycin (100 µg/ml) (Sigma-Aldrich, Steinheim, Germany) and sodium pyruvate (1 mM) (Invitrogen™, Paisley, UK). Primary BT cells were obtained from bovine testis collected in a local cattle slaughterhouse (Famalicão, Portugal) using methods previously reported [48] with some modifications. Briefly, testicles from bovine fetuses recovered at an abattoir from pregnant cows, were excised and the capsule was removed. The tissue was chopped with sterile scissors into pieces approximately 1 mm^3^ and washed with phosphate buffered saline (PBS) until rinse water is clear. The chopped tissue was added to a flask with solution of 0.25% trypsin (Sigma-Aldrich, Steinheim, Germany) at 37°C, a magnetic stirring bar and mixed at low speed for 10 min. New 0.25% trypsin solutions were added until large digested tissue fragments settle in the flask. Final digest was filtered through three layers sterile gauze to an iced flask for 3 times and centrifuged for 10 min at 6000xg. Supernatant was discarded and cells were resuspended in D-MEM (Invitrogen™, Paisley, UK) supplemented with 10% FBS (Invitrogen™, Paisley, UK), penicillin (100 U/ml) – streptomycin (100 µg/ml) (Sigma-Aldrich, Steinheim, Germany) at 37°C, 5% CO_2_. BT cells were maintained in the same conditions or stored in liquid nitrogen. Suspensions of liver homogenate samples (1 ml) filtered at 0.2 µm (Sarstedt, Nümbrecht, Germany) were inoculated on both cell cultures and these were subcultured once a week up to 7 and 2 passages of MDBK and BT cultures respectively. Uninoculated MDBK and BT cell cultures were also maintained in all passages as negative controls for all procedures.

### Immunofluorescence Assay

Immunofluorescence evaluation of HCV NS3, NS4A and NS5 specific proteins in MDBK and BT cells was performed as previously described [21], with some modifications. Cells inoculated with liver homogenate samples and the negative controls (uninoculated MDBK and BT cells) were subcultured 14 days post inoculation (second passage) and a drop of cell suspensions was placed on glass slides dots (6 mm) (Poly Labo, Strasbourg, France), pretreated with poly-L-lysine solution (Sigma-Aldrich, Steinheim, Germany). After incubation during 4 hours at 37°C, 5% CO_2_, the slides were fixed with ice-cold acetone (Merck, Darmstadt, Germany) and then treated with a blocking solution for 30 min. The slides were then incubated with mouse MAbs anti-HCV NS3, anti-HCV NS4A and anti-HCV NS5 (all from Santa Cruz Biotechnology, Inc, Heidelberg, Germany) (1∶100) in blocking solution at room temperature for 60 min, and subsequently incubated with goat anti-mouse IgG-FITC (Santa Cruz Biotechnology, Inc, Heidelberg, Germany) (1∶200) for 30 min. The slides were then counterstained with Evans blue (Sigma-Aldrich, Steinheim, Germany) for 5 min, washed 3 times with 1x PBS, mounted with florescence mounting solution and visualized in a fluorescence microscope (BX51, equipped with a DP72 camera and software Cell-B, Olympus, Tokyo, Japan).

### Immunogold Electron Microscopy

For IEM, 20 µl of cell suspensions at 14 days post inoculation from each MDBK cell flasks with the liver samples and the negative controls (uninoculated cells) were processed according to procedures previously described [22], with some modifications. In brief, cells were fixed in 1.25% glutaraldehyde (Electron Microscopy sciences, Hatfield, USA) and 2% paraformaldehyde (Merck, Darmstadt, Germany) in Tris Buffered Saline (TBS), dehydrated and embedded in Epon resin (TAAB, Berks, England). Ultrathin sections cut and placed on nickel grids (TAAB, Berks, England) and were floated for 10 min on a drop of TBS in a moist chamber. The grids were then allowed to float for 30 min on a drop of 14.4% sodium metaperiodate (Sigma-Aldrich, Steinheim, Germany) and then washed with TBS. After blocking with 2% BSA (Sigma-Aldrich, Steinheim, Germany) in TBS the grids were incubated for 60 min on a drop of the same primary MAbs used in IFA procedure diluted 1∶50 in TBS-BSA overnight at 4°C and then washed for several times. Then grids were incubated for 60 min in a drop of secondary anti-mouse IgG gold antibody (Sigma-Aldrich, Steinheim, Germany) diluted 1∶20 in TBS-BSA and then washed with TBS. Grids were counterstained with uranyl acetate and lead citrate as Reynolds [49] methods and visualized in a transmission electron microscope operated at 60 kV by Jeol 1400 (Tokyo, Japan) with a CCD digital camera Orious 1100W Tokyo, Japan at the Institute for Molecular and Cell Biology (IBMC) of the University of Porto, HEMS.

## Supporting Information

Figure S1 **PCR (A, C, E, and G) and RT-PCR (B, D, F, and H) generated amplicons of the HCV genomic regions from liver samples.** (A, B) Core protein; (C, D) Envelope proteins E1/E2; (E, F) NS5 A/B proteins and (G, H) RdRp-NS5B protein in domestic (1DR to 6DR); wild (1WR to 6WR) rabbits and hare (H). RT-PCR (I, J) generated amplicons of NS5 A/B protein in domestic rabbit serum (1DRS to 5DRS), domestic rabbit buffy coat (1DRB to 5DRB), wild rabbit’s body fluids (1WRF to 6WRF) and hare body fluids (HF). Negative control (NC); Marker 5 (Eurogentec, Seraing, Belgium) (M).
(TIF)

Figure S2 **MALDI-TOF/TOF-MS/MS spectra of the peptide sequences identified with HCV homolog fragments on liver samples.** Domestic rabbits (1–16), wild rabbits (17–28) and hare (29–34). Da – Dalton; C. I. – confidence interval; STNPKPQR (1), SQPRGRR (2), TWAQPGYPWPLYGNEGMGWAGWLLSPR (3), NSSIPTTTIRR (4), VASSTQSLVSWLSQGPSQK (5), SIEEFR (6), KVAGGHYVQMAFMK (7), GPITQMYTNVDQDLVGWPAPPGAR (8), AVDFIPVESLETTMR (9), DVWDWICTVLSDFKTWLQSKLLPR (10), IPGIPFISCQAGYR (11), NGSMRLAGPR (12), GSPPSLASSSASQLSAPSLK (13), VEFLVNTWK (14), AAIRSLTQR (15), AFTEAMTR (16), SQPRGRR (17), CDELAGKLKSLGLNAVAFYR (18), GRLGVYR (19), AKAPPPSWDAMWKCLAR (20), NGSMRISGSR (21), IVGPKMCSNVWNNR (22), VGDFHYVTGMTTDNVK (23), GSPPSLASSSASQLSAPSLK (24), SDLEPSIPSEYMLPKKR (25), SASLRQK (26), LLTVEEACALTPPHSAK (27), MALYDVTR (28), GSRPTWGPSDPRHR (29), LWHYPCTVNFTIFKVR (30), LGKEVLLGPADDYR (31), NGSMRLAGPR (32), AASKVSAR (33), DVRSHTSK (34). For more detailed information refer to Table 2.
(DOC)

Figure S3 **MALDI-TOF/TOF-MS/MS analysis of European rabbit and** ***Lepus europaeus*** **liver homogenates.** The peptides that match core, envelope glycoprotein’s E1and E2, protease NS2–3, serine protease NS3, NS5A, RdRp-NS5B HCV structural and non-structural proteins, with the reference strain HCV genotype 1b (Swiss-Prot/UniProt accession number POLG_HCVJA), are shown in boldface and underlined. Peptide sequences cover 12% (351/3010 aa) of the total genome polyprotein of the selected reference strain. Core (A) (positions 2–19), the matched peptides cover 28% (53/190 aa) of the protein. E1 (B) (positions 192–383), the matched peptides cover 6% (11/192 aa) of the protein. E2 (C) (positions 384–746), the matched peptides cover 9% (34/363 aa) of the protein. NS2–3 (D) (positions 810–1026), the matched peptides cover 22% (47/217 aa) of the protein. NS3 (E) (positions 1027–1657), the matched peptides cover 12% (73/631 aa) of the protein. NS5A (F) (positions 1973–2419), the matched peptides cover 17% (74/447 aa) of the protein. NS5B (G) (positions 2420–3010), the matched peptides cover 10% (59/591 aa) of the protein.
(TIFF)

Table S1 Homolog core HCV genomic fragment present in the European rabbit and *Lepus europaeus* genomes.
(DOC)

Table S2 Homolog E1/E2 HCV genomic fragment present in the European rabbit and *Lepus europaeus* genomes.
(DOC)

Table S3 Homolog NS5B HCV genomic fragment present in the European rabbit and *Lepus europaeus* genomes.
(DOC)

Table S4 Blastn between HCV 1b, *O. cuniclus* and *H. sapiens*.
(DOC)

Table S5 All HCV homolog proteins identified by MALDI-TOF/TOF-MS/MS analysis from the European rabbit and *Lepus europaeus* liver homogenates.
(DOC)

Table S6 Blastn between HCV specific primers and *O. cuniclus*.
(DOC)
