# Supplementary material for: Endogenous Hepatitis C Virus Homolog Fragments in European Rabbit and Hare Genomes Replicate in Cell Culture
Source: PLoS One. 2012 Nov 19;7(11):e49820. doi: 10.1371/journal.pone.0049820 (PMC3501476; doi:10.1371/journal.pone.0049820)
Supplement: Figure S2 — MALDI-TOF/TOF-MS/MS spectra of the peptide sequences identified with HCV homolog fragments on liver samples. Domestic rabbits (1–16), wild rabbits (17–28) and hare (29–34). Da – Dalton; C. I. – confidence interval; STNPKPQR (1), SQPRGRR (2), TWAQPGYPWPLYGNEGMGWAGWLLSPR (3), NSSIPTTTIRR (4), VASSTQSLVSWLSQGPSQK (5), SIEEFR (6), KVAGGHYVQMAFMK (7), GPITQMYTNVDQDLVGWPAPPGAR (8), AVDFIPVESLETTMR (9), DVWDWICTVLSDFKTWLQSKLLPR (10), IPGIPFISCQAGYR (11), NGSMRLAGPR (12), GSPPSLASSSASQLSAPSLK (13), VEFLVNTWK (14), AAIRSLTQR (15), AFTEAMTR (16), SQPRGRR (17), CDELAGKLKSLGLNAVAFYR (18), GRLGVYR (19), AKAPPPSWDAMWKCLAR (20), NGSMRISGSR (21), IVGPKMCSNVWNNR (22), VGDFHYVTGMTTDNVK (23), GSPPSLASSSASQLSAPSLK (24), SDLEPSIPSEYMLPKKR (25), SASLRQK (26), LLTVEEACALTPPHSAK (27), MALYDVTR (28), GSRPTWGPSDPRHR (29), LWHYPCTVNFTIFKVR (30), LGKEVLLGPADDYR (31), NGSMRLAGPR (32), AASKVSAR (33), DVRSHTSK (34). For more detailed information refer to Table 2. (DOC) [file pone.0049820.s002.doc]

**1**

| **Animal (specie)** | **Accession no. (genotype)** | **Protein name** | **Best peptide sequence** | **Observed mass (Da)** | **Total ion score C.I. %** | **E-value** |
| --- | --- | --- | --- | --- | --- | --- |
| DR(*O. cuniculus*) | POLG_HCVJA (1b) | Core protein | STNPKPQR | 927.48 | 99 | 0.39 |

**2**

| **Animal (specie)** | **Accession no. (genotype)** | **Protein name** | **Best peptide sequence** | **Observed mass (Da)** | **Total ion score C.I. %** | **E-value** |
| --- | --- | --- | --- | --- | --- | --- |
| DR(*O. cuniculus*) | POLG_HCVJA (1b) | Core protein | SQPRGRR | 856.47 | 99 | 0.23 |

**3**

| **Animal (specie)** | **Accession no. (genotype)** | **Protein name** | **Best peptide sequence** | **Observed mass (Da)** | **Total ion score C.I. %** | **E-value** |
| --- | --- | --- | --- | --- | --- | --- |
| DR(*O. cuniculus*) | POLG_HCVJA (1b) | Core protein | TWAQPGYPWPLYGNEGMGWAGWLLSPR | 3,090.54 | 99 | 0.3 |

**4**

| **Animal (specie)** | **Accession no. (genotype)** | **Protein name** | **Best peptide sequence** | **Observed mass (Da)** | **Total ion score C.I. %** | **E-value** |
| --- | --- | --- | --- | --- | --- | --- |
| DR(*O. cuniculus*) | POLG_HCVJA (1b) | Envelope glycoprotein E1 | NSSIPTTTIRR | 1,245.65 | 99 | 0.022 |

**5**

| **Animal (specie)** | **Accession no. (genotype)** | **Protein name** | **Best peptide sequence** | **Observed mass (Da)** | **Total ion score C.I. %** | **E-value** |
| --- | --- | --- | --- | --- | --- | --- |
| DR(*O. cuniculus*) | POLG_HCVJA (1b) | Envelope glycoprotein E2 | VASSTQSLVSWLSQGPSQK | 1,989.95 | 98 | 0.046 |

**6**

| **Animal (specie)** | **Accession no. (genotype)** | **Protein name** | **Best peptide sequence** | **Observed mass (Da)** | **Total ion score C.I. %** | **E-value** |
| --- | --- | --- | --- | --- | --- | --- |
| DR(*O. cuniculus*) | POLG_HCVVA (2K) | Envelope glycoprotein E2 | SIEEFR | 780.42 | 71 | 0.58 |

**7**

| **Animal (specie)** | **Accession no. (genotype)** | **Protein name** | **Best peptide sequence** | **Observed mass (Da)** | **Total ion score C.I. %** | **E-value** |
| --- | --- | --- | --- | --- | --- | --- |
| DR(*O. cuniculus*) | POLG_HCVBK (1b) | Protease NS2-3 | KVAGGHYVQMAFMK | 1,598.74 | 95 | 0.047 |

**8**

| **Animal (specie)** | **Accession no. (genotype)** | **Protein name** | **Best peptide sequence** | **Observed mass (Da)** | **Total ion score C.I. %** | **E-value** |
| --- | --- | --- | --- | --- | --- | --- |
| DR(*O. cuniculus*) | POLG_HCVJA (1b) | Serine protease NS3 | GPITQMYTNVDQDLVGWPAPPGAR | 2,583.32 | 81 | 0.6 |

**9**

| **Animal (specie)** | **Accession no. (genotype)** | **Protein name** | **Best peptide sequence** | **Observed mass (Da)** | **Total ion score C.I. %** | **E-value** |
| --- | --- | --- | --- | --- | --- | --- |
| DR(*O. cuniculus*) | POLG_HCVJ1 (1b) | Serine protease NS3 | AVDFIPVESLETTMR | 1,707.79 | 87 | 0.049 |

**10**

| **Animal (specie)** | **Accession no. (genotype)** | **Protein name** | **Best peptide sequence** | **Observed mass (Da)** | **Total ion score C.I. %** | **E-value** |
| --- | --- | --- | --- | --- | --- | --- |
| DR(*O. cuniculus*) | POLG_HCVJA (1b) | Non-structural protein 5A | DVWDWICTVLSDFKTWLQSKLLPR | 3,006.65 | 81 | 0.013 |

**11**

| **Animal (specie)** | **Accession no. (genotype)** | **Protein name** | **Best peptide sequence** | **Observed mass (Da)** | **Total ion score C.I. %** | **E-value** |
| --- | --- | --- | --- | --- | --- | --- |
| DR(*O. cuniculus*) | POLG_HCVT5 (6b) | Non-structural protein 5A | IPGIPFISCQAGYR | 1,578.80 | 89 | 0.15 |

**12**

| **Animal (specie)** | **Accession no. (genotype)** | **Protein name** | **Best peptide sequence** | **Observed mass (Da)** | **Total ion score C.I. %** | **E-value** |
| --- | --- | --- | --- | --- | --- | --- |
| DR(*O. cuniculus*) | POLG_HCVK3 (3a) | Non-structural protein 5A | NGSMRLAGPR | 1,074.53 | 74 | 0.92 |

**13**

| **Animal (specie)** | **Accession no. (genotype)** | **Protein name** | **Best peptide sequence** | **Observed mass (Da)** | **Total ion score C.I. %** | **E-value** |
| --- | --- | --- | --- | --- | --- | --- |
| DR(*O. cuniculus*) | POLG_HCVSA (5a) | Non-structural protein 5A | GSPPSLASSSASQLSAPSLK | 1,871.89 | 90 | 0.087 |

**14**

| **Animal (specie)** | **Accession no. (genotype)** | **Protein name** | **Best peptide sequence** | **Observed mass (Da)** | **Total ion score C.I. %** | **E-value** |
| --- | --- | --- | --- | --- | --- | --- |
| DR(*O. cuniculus*) | POLG_HCVJA (1b) | RdRp-NS5B | VEFLVNTWK | 1,135.62 | 81 | 0.15 |

**15**

| **Animal (specie)** | **Accession no. (genotype)** | **Protein name** | **Best peptide sequence** | **Observed mass (Da)** | **Total ion score C.I. %** | **E-value** |
| --- | --- | --- | --- | --- | --- | --- |
| DR(*O. cuniculus*) | POLG_HCVSA (5a) | RdRp-NS5B | AAIRSLTQR | 1,015.58 | 99 | 0.1 |

**16**

| **Animal (specie)** | **Accession no. (genotype)** | **Protein name** | **Best peptide sequence** | **Observed mass (Da)** | **Total ion score C.I. %** | **E-value** |
| --- | --- | --- | --- | --- | --- | --- |
| DR(*O. cuniculus*) | POLG_HCVJA (1b) | RdRp-NS5B | AFTEAMTR | 926.43 | 81 | 1.1 |

**17**

| **Animal (specie)** | **Accession no. (genotype)** | **Protein name** | **Best peptide sequence** | **Observed mass (Da)** | **Total ion score C.I. %** | **E-value** |
| --- | --- | --- | --- | --- | --- | --- |
| WR(*O. cuniculus*) | POLG_HCVNZ (3a) | Core protein | SQPRGRR | 856.52 | 22 | 1.1 |

**18**

| **Animal (specie)** | **Accession no. (genotype)** | **Protein name** | **Best peptide sequence** | **Observed mass (Da)** | **Total ion score C.I. %** | **E-value** |
| --- | --- | --- | --- | --- | --- | --- |
| WR(*O. cuniculus*) | POLG_HCV6A (6a ) | Serine protease NS3 | CDELAGKLKSLGLNAVAFYR | 2,225.13 | 87 | 0.15 |

**19**

| **Animal (specie)** | **Accession no. (genotype)** | **Protein name** | **Best peptide sequence** | **Observed mass (Da)** | **Total ion score C.I. %** | **E-value** |
| --- | --- | --- | --- | --- | --- | --- |
| WR(*O. cuniculus*) | POLG_HCVJ8 (2b)) | Serine protease NS3 | GRLGVYR | 820.44 | 57 | 0.05 |

**20**

| **Animal (specie)** | **Accession no. (genotype)** | **Protein name** | **Best peptide sequence** | **Observed mass (Da)** | **Total ion score C.I. %** | **E-value** |
| --- | --- | --- | --- | --- | --- | --- |
| WR(*O. cuniculus*) | POLG_HCVJF (2a) | Serine protease NS3 | AKAPPPSWDAMWKCLAR | 2,001.02 | 93 | 0.016 |

**21**

| **Animal (specie)** | **Accession no. (genotype)** | **Protein name** | **Best peptide sequence** | **Observed mass (Da)** | **Total ion score C.I. %** | **E-value** |
| --- | --- | --- | --- | --- | --- | --- |
| WR(*O. cuniculus*) | POLG_HCVVO (6K) | Non-structural protein 5A | NGSMRISGSR | 1,064.57 | 2293 | 0.05 |

**22**

| **Animal (specie)** | **Accession no. (genotype)** | **Protein name** | **Best peptide sequence** | **Observed mass (Da)** | **Total ion score C.I. %** | **E-value** |
| --- | --- | --- | --- | --- | --- | --- |
| WR(*O. cuniculus*) | POLG_HCVVN (6d) | Non-structural protein 5A | IVGPKMCSNVWNNR | 1,690.83 | 87 | 0.2 |

**23**

| **Animal (specie)** | **Accession no. (genotype)** | **Protein name** | **Best peptide sequence** | **Observed mass (Da)** | **Total ion score C.I. %** | **E-value** |
| --- | --- | --- | --- | --- | --- | --- |
| WR(*O. cuniculus*) | POLG_HCVCO (1b) | Non-structural protein 5A | VGDFHYVTGMTTDNVK | 1,799.89 | 74 | 0.5 |

**24**

| **Animal (specie)** | **Accession no. (genotype)** | **Protein name** | **Best peptide sequence** | **Observed mass (Da)** | **Total ion score C.I. %** | **E-value** |
| --- | --- | --- | --- | --- | --- | --- |
| WR(*O. cuniculus*) | POLG_HCVCO (1b) | Non-structural protein 5A | GSPPSLASSSASQLSAPSLK | 1,871.89 | 74 | 0.53 |

**25**

| **Animal (specie)** | **Accession no. (genotype)** | **Protein name** | **Best peptide sequence** | **Observed mass (Da)** | **Total ion score C.I. %** | **E-value** |
| --- | --- | --- | --- | --- | --- | --- |
| WR(*O. cuniculus*) | POLG_HCVJ6 (2a) | Non-structural protein 5A | SDLEPSIPSEYMLPKKR | 1,989.98 | 22 | 0.1 |

**26**

| **Animal (specie)** | **Accession no. (genotype)** | **Protein name** | **Best peptide sequence** | **Observed mass (Da)** | **Total ion score C.I. %** | **E-value** |
| --- | --- | --- | --- | --- | --- | --- |
| WR(*O. cuniculus*) | POLG_HCV6A (6a) | RdRp-NS5B | SASLRQK | 789.46 | 87 | 0.13 |

**27**

| **Animal (specie)** | **Accession no. (genotype)** | **Protein name** | **Best peptide sequence** | **Observed mass (Da)** | **Total ion score C.I. %** | **E-value** |
| --- | --- | --- | --- | --- | --- | --- |
| WR(*O. cuniculus*) | POLG_HCVJ8 (2b) | RdRp-NS5B | LLTVEEACALTPPHSAK | 1,836.90 | 57 | 0.15 |

**28**

| **Animal (specie)** | **Accession no. (genotype)** | **Protein name** | **Best peptide sequence** | **Observed mass (Da)** | **Total ion score C.I. %** | **E-value** |
| --- | --- | --- | --- | --- | --- | --- |
| WR(*O. cuniculus*) | POLG_HCV6A (6a) | RdRp-NS5B | MALYDVTR | 984.49 | 87 | 0.2 |

**29**

| **Animal (specie)** | **Accession no. (genotype)** | **Protein name** | **Best peptide sequence** | **Observed mass (Da)** | **Total ion score C.I. %** | **E-value** |
| --- | --- | --- | --- | --- | --- | --- |
| *Hare(Lepus europaeus)* | POLG_HCVJP (2b) | Core protein | GSRPTWGPSDPRHR | 1,605.84 | 53 | 0.13 |

**30**

| **Animal (specie)** | **Accession no. (genotype)** | **Protein name** | **Best peptide sequence** | **Observed mass (Da)** | **Total ion score C.I. %** | **E-value** |
| --- | --- | --- | --- | --- | --- | --- |
| *Hare(Lepus europaeus)* | POLG_HCVJP (2b) | Envelope glycoprotein E2 | LWHYPCTVNFTIFKVR | 2,081.04 | 53 | 0.65 |

**31**

| **Animal (specie)** | **Accession no. (genotype)** | **Protein name** | **Best peptide sequence** | **Observed mass (Da)** | **Total ion score C.I. %** | **E-value** |
| --- | --- | --- | --- | --- | --- | --- |
| *Hare(Lepus europaeus)* | POLG_HCVJK (3K) | Protease NS2-3 | LGKEVLLGPADDYR | 1,545.76 | 81 | 0.067 |

**32**

| **Animal (specie)** | **Accession no. (genotype)** | **Protein name** | **Best peptide sequence** | **Observed mass (Da)** | **Total ion score C.I. %** | **E-value** |
| --- | --- | --- | --- | --- | --- | --- |
| *Hare(Lepus europaeus)* | POLG_HCVK3 (3a) | Non-structural protein 5A | NGSMRLAGPR | 1,074.50 | 67 | 0.92 |

**33**

| **Animal (specie)** | **Accession no. (genotype)** | **Protein name** | **Best peptide sequence** | **Observed mass (Da)** | **Total ion score C.I. %** | **E-value** |
| --- | --- | --- | --- | --- | --- | --- |
| *Hare(Lepus europaeus)* | POLG_HCVJP (2b) | RdRp-NS5B | AASKVSAR | 789.46 | 53 | 0.65 |

**34**

| **Animal (specie)** | **Accession no. (genotype)** | **Protein name** | **Best peptide sequence** | **Observed mass (Da)** | **Total ion score C.I. %** | **E-value** |
| --- | --- | --- | --- | --- | --- | --- |
| *Hare(Lepus europaeus)* | POLG_HCVT5 (6b) | RdRp-NS5B | DVRSHTSK | 929.52 | 82 | 0.09 |
