# Supplementary material for: Endogenous Hepatitis C Virus Homolog Fragments in European Rabbit and Hare Genomes Replicate in Cell Culture
Source: PLoS One. 2012 Nov 19;7(11):e49820. doi: 10.1371/journal.pone.0049820 (PMC3501476; doi:10.1371/journal.pone.0049820)
Supplement: Table S1 — Homolog core HCV genomic fragment present in the European rabbit and Lepus europaeus genomes. (DOC) [file pone.0049820.s004.doc]

**Table S1.** Homolog core HCV genomic fragment present in the European rabbit and *Lepus europaeus* genomes.

| **Accession no.** | **Name** | **Genotype** | **Country** | **Year** | **Description** | **Genome position (nt)^1^** | **Score (bits)** | **E-value** | **Identities** |
| --- | --- | --- | --- | --- | --- | --- | --- | --- | --- |
| [FR850665](http://hcv.lanl.gov/components/sequence/HCV/asearch/query_one.comp?se_id=132450&accession=FR850665) | RO-IDU8/2008 | 1b | RO | 2008 | Hepatitis C virus subtype 1b partial gene for polyprotein, core protein region, isolate RO-IDU8/2008 | 208-188 | 35 | 0.005 | 21/21(100%) |
| [FR850667](http://hcv.lanl.gov/components/sequence/HCV/asearch/query_one.comp?se_id=132448&accession=FR850667) | RO-IDU10/2008 | 1b | RO | 2008 | Hepatitis C virus subtype 1b partial gene for polyprotein, core protein region, isolate RO-IDU10/2008 | 208-188 | 35 | 0.005 | 21/21(100%) |
| [FR850670](http://hcv.lanl.gov/components/sequence/HCV/asearch/query_one.comp?se_id=132445&accession=FR850670) | RO-IDU13/2008 | 1a | RO | 2008 | Hepatitis C virus subtype 1a partial gene for polyprotein, core protein region, isolate RO-IDU13/2008 | 208-188 | 35 | 0.005 | 21/21(100%) |
| [FR850671](http://hcv.lanl.gov/components/sequence/HCV/asearch/query_one.comp?se_id=132444&accession=FR850671) | RO-IDU14/2008 | 1a | RO | 2008 | Hepatitis C virus subtype 1a partial gene for polyprotein, core protein region, isolate RO-IDU14/2008 | 211-191 | 35 | 0.005 | 21/21(100%) |
| [FR850672](http://hcv.lanl.gov/components/sequence/HCV/asearch/query_one.comp?se_id=132443&accession=FR850672) | RO-IDU15/2008 | 1b | RO | 2008 | Hepatitis C virus subtype 1b partial gene for polyprotein, core protein region, isolate RO-IDU15/2008 | 208-188 | 35 | 0.005 | 21/21(100%) |
| [FR850673](http://hcv.lanl.gov/components/sequence/HCV/asearch/query_one.comp?se_id=132442&accession=FR850673) | RO-IDU16/2008 | 1b | RO | 2008 | Hepatitis C virus subtype 1b partial gene for polyprotein, core protein region, isolate RO-IDU16/2008 | 211-191 | 35 | 0.005 | 21/21(100%) |
| [HQ729760](http://hcv.lanl.gov/components/sequence/HCV/asearch/query_one.comp?se_id=132220&accession=HQ729760) | N21 | 1a | SE | - | Hepatitis C virus isolate N21 polyprotein gene, partial cds | 169-149 | 35 | 0.005 | 21/21(100%) |
| [HM106537](http://hcv.lanl.gov/components/sequence/HCV/asearch/query_one.comp?se_id=128700&accession=HM106537) | 1545 | 1b | IE | 2001 | Hepatitis C virus isolate 1545 core protein gene, partial cds | 169-149 | 35 | 0.005 | 21/21(100%) |
| [HM106543](http://hcv.lanl.gov/components/sequence/HCV/asearch/query_one.comp?se_id=128694&accession=HM106543) | 1554 | 1b | IE | 2002 | Hepatitis C virus isolate 1554 core protein gene, partial cds | 169-149 | 35 | 0.005 | 21/21(100%) |
| [FR854392](http://hcv.lanl.gov/components/sequence/HCV/asearch/query_one.comp?se_id=128255&accession=FR854392) | RO-IDU1/2008 | 1a | RO | 2008 | Hepatitis C virus partial gene for polyprotein, core protein region, genomic RNA, isolate RO-IDU1/2008 | 211-191 | 35 | 0.005 | 21/21(100%) |
| [EU484142](http://hcv.lanl.gov/components/sequence/HCV/asearch/query_one.comp?se_id=126534&accession=EU484142) | AV77 | 1 | IN | - | Hepatitis C virus genotype 1 isolate AV77 polyprotein gene, partial cds | 123-103 | 35 | 0.005 | 21/21(100%) |
| [EU484143](http://hcv.lanl.gov/components/sequence/HCV/asearch/query_one.comp?se_id=126533&accession=EU484143) | AV78 | 1 | IN | - | Hepatitis C virus genotype 1 isolate AV78 nonfunctional polyprotein gene, partial sequence | 121-101 | 35 | 0.005 | 21/21(100%) |
| [EU484144](http://hcv.lanl.gov/components/sequence/HCV/asearch/query_one.comp?se_id=126532&accession=EU484144) | AV79 | 1 | IN | - | Hepatitis C virus genotype 1 isolate AV79 polyprotein gene, partial cds | 140-120 | 35 | 0.005 | 21/21(100%) |
| [HQ912956](http://hcv.lanl.gov/components/sequence/HCV/asearch/query_one.comp?se_id=125913&accession=HQ912956) | PR26 | 1b | CN | 2007 | Hepatitis C virus subtype 1b isolate PR26 polyprotein gene, complete cds | 169-149 | 35 | 0.005 | 21/21(100%) |
| [HQ912958](http://hcv.lanl.gov/components/sequence/HCV/asearch/query_one.comp?se_id=125911&accession=HQ912958) | PR52 | 1b | CN | 2007 | Hepatitis C virus subtype 1b isolate PR52 polyprotein gene, complete cds | 169-149 | 35 | 0.005 | 21/21(100%) |
| [HQ912959](http://hcv.lanl.gov/components/sequence/HCV/asearch/query_one.comp?se_id=125910&accession=HQ912959) | PR79 | 1b | CN | 2008 | Hepatitis C virus subtype 1b isolate PR79 polyprotein gene, complete cds | 169-149 | 35 | 0.005 | 21/21(100%) |
| [GU186929](http://hcv.lanl.gov/components/sequence/HCV/asearch/query_one.comp?se_id=119982&accession=GU186929) | CBD413 | 1b | KH | 2007 | Hepatitis C virus isolate CBD413 polyprotein gene, partial cds | 178-158 | 35 | 0.005 | 21/21(100%) |
| [GU186936](http://hcv.lanl.gov/components/sequence/HCV/asearch/query_one.comp?se_id=119975&accession=GU186936) | MM1038 | 1a | MM | 2008 | Hepatitis C virus isolate MM1038 polyprotein gene, partial cds | 190-170 | 35 | 0.005 | 21/21(100%) |
| [FN675983](http://hcv.lanl.gov/components/sequence/HCV/asearch/query_one.comp?se_id=119853&accession=FN675983) | 3906 | 1b | ES | - | Hepatitis C virus subtype 1b partial gene for polyprotein, isolate 3906, genomic RNA | 169-149 | 35 | 0.005 | 21/21(100%) |
| [FN675945](http://hcv.lanl.gov/components/sequence/HCV/asearch/query_one.comp?se_id=119835&accession=FN675945) | 1313 | 1b | ES | - | Hepatitis C virus subtype 1b partial gene for polyprotein, isolate 1313, genomic RNA | 169-149 | 35 | 0.005 | 21/21(100%) |
| [FN675947](http://hcv.lanl.gov/components/sequence/HCV/asearch/query_one.comp?se_id=119833&accession=FN675947) | 1616 | 1b | ES | - | Hepatitis C virus subtype 1b partial gene for polyprotein, isolate 1616, genomic RNA | 169-149 | 35 | 0.005 | 21/21(100%) |
| [FN675951](http://hcv.lanl.gov/components/sequence/HCV/asearch/query_one.comp?se_id=119829&accession=FN675951) | 1955 | 1b | ES | 1955 | Hepatitis C virus subtype 1b partial gene for polyprotein, isolate 1955, genomic RNA | 169-149 | 35 | 0.005 | 21/21(100%) |
| [FN675956](http://hcv.lanl.gov/components/sequence/HCV/asearch/query_one.comp?se_id=119824&accession=FN675956) | 3249 | 1b | ES | - | Hepatitis C virus subtype 1b partial gene for polyprotein, isolate 3249, genomic RNA | 169-149 | 35 | 0.005 | 21/21(100%) |
| [HH731927](http://hcv.lanl.gov/components/sequence/HCV/asearch/query_one.comp?se_id=117686&accession=HH731927) | HH731927 | - | - | - | Sequence 6691 from Patent EP2216407 | 510-490 | 35 | 0.005 | 21/21(100%) |
| [AB518823](http://hcv.lanl.gov/components/sequence/HCV/asearch/query_one.comp?se_id=117409&accession=AB518823) | PAT.44 | 1b | - | - | Hepatitis C virus subtype 1b gene for polyprotein, core protein region, partial cds, isolate: PAT.44 | 169-149 | 35 | 0.005 | 21/21(100%) |
| [AB518836](http://hcv.lanl.gov/components/sequence/HCV/asearch/query_one.comp?se_id=117396&accession=AB518836) | PAT.73 | 1b | - | - | Hepatitis C virus subtype 1b gene for polyprotein, core protein region, partial cds, isolate: PAT.73 | 169-149 | 35 | 0.005 | 21/21(100%) |
| [AB518837](http://hcv.lanl.gov/components/sequence/HCV/asearch/query_one.comp?se_id=117395&accession=AB518837) | PAT.81 | 1b | - | - | Hepatitis C virus subtype 1b gene for polyprotein, core protein region, partial cds, isolate: PAT.81 | 121-101 | 35 | 0.005 | 21/21(100%) |
| [AB518856](http://hcv.lanl.gov/components/sequence/HCV/asearch/query_one.comp?se_id=117376&accession=AB518856) | PAT.135 | 1b | - | - | Hepatitis C virus subtype 1b gene for polyprotein, core protein region, partial cds, isolate: PAT.135 | 169-149 | 35 | 0.005 | 21/21(100%) |
| [GU441256](http://hcv.lanl.gov/components/sequence/HCV/asearch/query_one.comp?se_id=113632&accession=GU441256) | GU441256 | 1b | ID | 2008 | Hepatitis C virus subtype 1b strain 08.40.039 core gene, partial cds | 304-284 | 35 | 0.005 | 21/21(100%) |
| [GU441257](http://hcv.lanl.gov/components/sequence/HCV/asearch/query_one.comp?se_id=113631&accession=GU441257) | GU441257 | 1b | ID | 2008 | Hepatitis C virus subtype 1b strain 08.40.052 core gene, partial cds | 304-284 | 35 | 0.005 | 21/21(100%) |
| [GU441282](http://hcv.lanl.gov/components/sequence/HCV/asearch/query_one.comp?se_id=113606&accession=GU441282) | GU441282 | 1b | ID | 2006 | Hepatitis C virus subtype 1b strain P.X00.74 core gene, partial cds | 304-284 | 35 | 0.005 | 21/21(100%) |
| [GU441297](http://hcv.lanl.gov/components/sequence/HCV/asearch/query_one.comp?se_id=113591&accession=GU441297) | GU441297 | 1b | ID | 2007 | Hepatitis C virus subtype 1b strain 07.10.122 core gene, partial cds | 304-284 | 35 | 0.005 | 21/21(100%) |
| [HM000538](http://hcv.lanl.gov/components/sequence/HCV/asearch/query_one.comp?se_id=113126&accession=HM000538) | 161v11c05_y00 | 1a | US | 2000 | Hepatitis C virus isolate 161v11c05_y00 polyprotein gene, partial cds | 455-435 | 35 | 0.005 | 21/21(100%) |
| [HM000539](http://hcv.lanl.gov/components/sequence/HCV/asearch/query_one.comp?se_id=113125&accession=HM000539) | 161v20c17_y01 | 1a | US | 2001 | Hepatitis C virus isolate 161v20c17_y01 polyprotein gene, partial cds | 441-421 | 35 | 0.005 | 21/21(100%) |
| [HM000540](http://hcv.lanl.gov/components/sequence/HCV/asearch/query_one.comp?se_id=113124&accession=HM000540) | 161v26c06_y02 | 1a | US | 2002 | Hepatitis C virus isolate 161v26c06_y02 polyprotein gene, partial cds | 440-420 | 35 | 0.005 | 21/21(100%) |
| [HM000541](http://hcv.lanl.gov/components/sequence/HCV/asearch/query_one.comp?se_id=113123&accession=HM000541) | 161v26c08_y02 | 1a | US | 2002 | Hepatitis C virus isolate 161v26c08_y02 polyprotein gene, partial cds | 441-421 | 35 | 0.005 | 21/21(100%) |
| [HM000542](http://hcv.lanl.gov/components/sequence/HCV/asearch/query_one.comp?se_id=113122&accession=HM000542) | 161v32c10_y03 | 1a | US | 2003 | Hepatitis C virus isolate 161v32c10_y03 polyprotein gene, partial cds | 441-421 | 35 | 0.005 | 21/21(100%) |
| [FJ483368](http://hcv.lanl.gov/components/sequence/HCV/asearch/query_one.comp?se_id=111989&accession=FJ483368) | patient 8 | 1b | JP | 2002 | Hepatitis C virus subtype 1b patient 8 polyprotein gene, partial cds | 169-149 | 35 | 0.005 | 21/21(100%) |
| [FJ483372](http://hcv.lanl.gov/components/sequence/HCV/asearch/query_one.comp?se_id=111985&accession=FJ483372) | patient 12 | 1b | JP | 2003 | Hepatitis C virus subtype 1b patient 12 polyprotein gene, partial cds | 169-149 | 35 | 0.005 | 21/21(100%) |
| [FJ483379](http://hcv.lanl.gov/components/sequence/HCV/asearch/query_one.comp?se_id=111978&accession=FJ483379) | patient 19 | 1b | JP | 2004 | Hepatitis C virus subtype 1b patient 19 polyprotein gene, partial cds | 169-149 | 35 | 0.005 | 21/21(100%) |
| [FJ483400](http://hcv.lanl.gov/components/sequence/HCV/asearch/query_one.comp?se_id=111957&accession=FJ483400) | patient 40 | 1b | JP | 2006 | Hepatitis C virus subtype 1b patient 40 polyprotein gene, partial cds | 169-149 | 35 | 0.005 | 21/21(100%) |
| [FN666322](http://hcv.lanl.gov/components/sequence/HCV/asearch/query_one.comp?se_id=111862&accession=FN666322) | patient 319 | 1a | GB | 2006 | Hepatitis C virus subtype 1a partial gene for polyprotein, core protein region, isolated from patient 319 | 169-149 | 35 | 0.005 | 21/21(100%) |
| [FJ411256](http://hcv.lanl.gov/components/sequence/HCV/asearch/query_one.comp?se_id=93338&accession=FJ411256) | 27 | 1b | RU | 2000 | Hepatitis C virus isolate 27 clone 1-6 polyprotein gene, partial cds | 486-466 | 35 | 0.005 | 21/21(100%) |
| [AY506668](http://hcv.lanl.gov/components/sequence/HCV/asearch/query_one.comp?se_id=56032&accession=AY506668) | Arg04 | 1b | AR | - | Hepatitis C virus type 1b isolate Arg04 core protein gene, partial cds | 50-30 | 35 | 0.005 | 21/21(100%) |
| [L38343](http://hcv.lanl.gov/components/sequence/HCV/asearch/query_one.comp?se_id=71790&accession=L38343) | BE101 | 1b | BE | - | Hepatitis C virus type 1b (clone BE102) core protein mRNA, 5' end of cds | 407-387 | 35 | 0.005 | 21/21(100%) |
| [AY506683](http://hcv.lanl.gov/components/sequence/HCV/asearch/query_one.comp?se_id=56017&accession=AY506683) | Arg27 | 1b | AR | - | Hepatitis C virus type 1b isolate Arg27 core protein gene, partial cds | 50-30 | 35 | 0.005 | 21/21(100%) |
| [AM494583](http://hcv.lanl.gov/components/sequence/HCV/asearch/query_one.comp?se_id=21994&accession=AM494583) | genotype-1b | 1b | CU | 2006 | Hepatitis C virus partial cp gene for capsid protein, isolate genotype 1b, clone HCV-Cu40 | 76-56 | 35 | 0.005 | 21/21(100%) |
| [DQ001225](http://hcv.lanl.gov/components/sequence/HCV/asearch/query_one.comp?se_id=46286&accession=DQ001225) | HIA293 | 1b | RU | - | Hepatitis C virus isolate HIA293 core protein gene, partial cds | 157-137 | 35 | 0.005 | 21/21(100%) |
| [S83169](http://hcv.lanl.gov/components/sequence/HCV/asearch/query_one.comp?se_id=40692&accession=S83169) | S83169 | 1b | - | - | Hepatitis C virus core protein gene, partial cds | 169-149 | 35 | 0.005 | 21/21(100%) |
| [AF506614](http://hcv.lanl.gov/components/sequence/HCV/asearch/query_one.comp?se_id=64963&accession=AF506614) | RIG260 | 1b | RU | - | Hepatitis C virus isolate RIG260 polyprotein precursor gene, core protein region, partial cds | 169-149 | 35 | 0.005 | 21/21(100%) |

Blast nucleotide homology between homolog core HCV fragment (**GTGACCGCTCGGAAGTCTTCC**) generated by PCR and RT-PCR of studied liver samples and HCV sequences deposited at the site <http://hcv.lanl.gov/content/sequence/BASIC_BLAST/basic_blast.html>. ^1^ Reverse complement. CA - Canada, RO - Romania, SE - Sweden, IE - Ireland, IN - India, CN - China, KH - Cambodia, MM - Myanmar, ES - Spain, ID - Indonesia, US - USA, JP - Japan, RU - Russia, AR - Argentina, CU - Cuba.
