# Supplementary material for: Endogenous Hepatitis C Virus Homolog Fragments in European Rabbit and Hare Genomes Replicate in Cell Culture
Source: PLoS One. 2012 Nov 19;7(11):e49820. doi: 10.1371/journal.pone.0049820 (PMC3501476; doi:10.1371/journal.pone.0049820)
Supplement: Table S2 — Homolog E1/E2 HCV genomic fragment present in the European rabbit and Lepus europaeus genomes. (DOC) [file pone.0049820.s005.doc]

**Table S2.** Homolog E1/E2 HCV genomic fragment present in the European rabbit and *Lepus europaeus* genomes.

| **Accessio no.** | **Name** | **Genotype** | **Country** | **Year** | **Description** | **Genome position (nt)** | **Score (bits)** | **E-value** | **Identities** |
| --- | --- | --- | --- | --- | --- | --- | --- | --- | --- |
| [AY767496](http://hcv.lanl.gov/components/sequence/HCV/asearch/query_one.comp?se_id=52238&accession=AY767496) | 38146-R | 3a | - | - | Hepatitis C virus isolate 38146-R core E1 gene, partial sequence | 133-158 | 30 | 0.12 | 22/26 (84%) |
| [DQ776883](http://hcv.lanl.gov/components/sequence/HCV/asearch/query_one.comp?se_id=17744&accession=DQ776883) | 3229 | 3a | CA | - | Hepatitis C virus isolate 3229 polyprotein gene, partial cds | 157-182 | 30 | 0.12 | 22/26 (84%) |
| [HQ318842](http://hcv.lanl.gov/components/sequence/HCV/asearch/query_one.comp?se_id=125428&accession=HQ318842) | 09CNJSZJ115 | 3a | CN | 2009 | Hepatitis C virus isolate 09CNJSZJ115 polyprotein gene, partial cds | 123-148 | 27 | 1.1 | 21/26 (80%) |
| [EF420128](http://hcv.lanl.gov/components/sequence/HCV/asearch/query_one.comp?se_id=13427&accession=EF420128) | R30 | 3 | NL | - | Hepatitis C virus strain R30 polyprotein gene, partial cds | 997-1022 | 27 | 1.1 | 21/26 (80%) |
| [DQ776865](http://hcv.lanl.gov/components/sequence/HCV/asearch/query_one.comp?se_id=17762&accession=DQ776865) | 3397 | 3a | CA | - | Hepatitis C virus isolate 3397 polyprotein gene, partial cds | 157-182 | 27 | 1.1 | 21/26 (80%) |
| [DQ776872](http://hcv.lanl.gov/components/sequence/HCV/asearch/query_one.comp?se_id=17755&accession=DQ776872) | 3017 | 3a | CA | - | Hepatitis C virus isolate 3017 polyprotein gene, partial cds | 157-182 | 27 | 1.1 | 21/26 (80%) |
| [EF185976](http://hcv.lanl.gov/components/sequence/HCV/asearch/query_one.comp?se_id=2548&accession=EF185976) | 4D | 3a | CN | - | Hepatitis C virus subtype 3a isolate 4D polyprotein gene, partial cds | 127-149 | 25 | 3.3 | 19/23 (82%) |
| [AY767819](http://hcv.lanl.gov/components/sequence/HCV/asearch/query_one.comp?se_id=51915&accession=AY767819) | 43833-R | 3a | - | - | Hepatitis C virus isolate 43833-R core E1 gene, partial sequence | 3-25 | 25 | 3.3 | 19/23 (82%) |
| [AY768116](http://hcv.lanl.gov/components/sequence/HCV/asearch/query_one.comp?se_id=51618&accession=AY768116) | 50241-R | 3a | - | - | Hepatitis C virus isolate 50241-R core E1 gene, partial sequence | 87-109 | 25 | 3.3 | 19/23 (82%) |
| [JF343784](http://hcv.lanl.gov/components/sequence/HCV/asearch/query_one.comp?se_id=132435&accession=JF343784) | S52-JFH1 | 3a/2a | DK | 2011 | Recombinant Hepatitis C virus S52(5'UTR-NS2)/JFH1_I793S, K1404Q, complete genome | 1023-1048 | 24 | 9.9 | 20/26 (76%) |
| [JA206597](http://hcv.lanl.gov/components/sequence/HCV/asearch/query_one.comp?se_id=132409&accession=JA206597) | JA206597 | - | - | - | Sequence 3 from Patent WO2011038737 | 1023-1048 | 24 | 9.9 | 20/26 (76%) |
| [JA212561](http://hcv.lanl.gov/components/sequence/HCV/asearch/query_one.comp?se_id=132351&accession=JA212561) | JA212561 | - | - | - | Sequence 49 from Patent WO2011039639 | 1024-1049 | 24 | 9.9 | 20/26 (76%) |
| [JA212562](http://hcv.lanl.gov/components/sequence/HCV/asearch/query_one.comp?se_id=132350&accession=JA212562) | JA212562 | - | - | - | Sequence 50 from Patent WO2011039639 | 1024-1049 | 24 | 9.9 | 20/26 (76%) |
| [EU204645](http://hcv.lanl.gov/components/sequence/HCV/asearch/query_one.comp?se_id=128009&accession=EU204645) | EU204645 | 3a/2a | DK | - | Recombinant Hepatitis C virus S52/JFH1, complete genome | 1024-1049 | 24 | 9.9 | 20/26 (76%) |
| [FN401009](http://hcv.lanl.gov/components/sequence/HCV/asearch/query_one.comp?se_id=126102&accession=FN401009) | 123C_PT | 3a | PT | - | Hepatitis C virus subtype 3a partial gene for polyprotein, core/E1 region, genomic RNA, isolate 123C_PT | 137-162 | 24 | 9.9 | 20/26 (76%) |
| [HQ318841](http://hcv.lanl.gov/components/sequence/HCV/asearch/query_one.comp?se_id=125429&accession=HQ318841) | 09CNJSZJ109 | 3a | CN | 2009 | Hepatitis C virus isolate 09CNJSZJ109 polyprotein gene, partial cds | 123-148 | 24 | 9.9 | 20/26 (76%) |
| [HQ318849](http://hcv.lanl.gov/components/sequence/HCV/asearch/query_one.comp?se_id=125421&accession=HQ318849) | 09CNJSZJ033 | 3a | CN | 2009 | Hepatitis C virus isolate 09CNJSZJ033 polyprotein gene, partial cds | 123-148 | 24 | 9.9 | 20/26 (76%) |
| [HQ318859](http://hcv.lanl.gov/components/sequence/HCV/asearch/query_one.comp?se_id=125411&accession=HQ318859) | 09CNJSZJ279 | 3a | CN | 2009 | Hepatitis C virus isolate 09CNJSZJ279 polyprotein gene, partial cds | 169-194 | 24 | 9.9 | 20/26 (76%) |
| [HQ108100](http://hcv.lanl.gov/components/sequence/HCV/asearch/query_one.comp?se_id=115960&accession=HQ108100) | S49 | 3a | PK | 2009 | Hepatitis C virus clone HCVPK9 polyprotein gene, partial cds | 684-709 | 24 | 9.9 | 20/26 (76%) |
| [HQ108101](http://hcv.lanl.gov/components/sequence/HCV/asearch/query_one.comp?se_id=115959&accession=HQ108101) | S49 | 3a | PK | 2009 | Hepatitis C virus clone HCVPK10 polyprotein gene, partial cds | 684-709 | 24 | 9.9 | 20/26 (76%) |
| [HQ108102](http://hcv.lanl.gov/components/sequence/HCV/asearch/query_one.comp?se_id=115958&accession=HQ108102) | S49 | 3a | PK | 2009 | Hepatitis C virus clone HCVPK11 polyprotein gene, partial cds | 684-709 | 24 | 9.9 | 20/26 (76%) |
| [HQ108103](http://hcv.lanl.gov/components/sequence/HCV/asearch/query_one.comp?se_id=115957&accession=HQ108103) | S49 | 3a | PK | 2009 | Hepatitis C virus clone HCVPK12 polyprotein gene, partial cds | 684-709 | 24 | 9.9 | 20/26 (76%) |
| [HC510398](http://hcv.lanl.gov/components/sequence/HCV/asearch/query_one.comp?se_id=113660&accession=HC510398) | HC510398 | - | - | - | Sequence 124 from Patent WO2010037403 | 1024-1049 | 24 | 9.9 | 20/26 (76%) |
| [HC510299](http://hcv.lanl.gov/components/sequence/HCV/asearch/query_one.comp?se_id=113709&accession=HC510299) | HC510299 | - | - | - | Sequence 25 from Patent WO2010037403 | 1023-1048 | 24 | 9.9 | 20/26 (76%) |
| [HC510314](http://hcv.lanl.gov/components/sequence/HCV/asearch/query_one.comp?se_id=113702&accession=HC510314) | HC510314 | - | - | - | Sequence 40 from Patent WO2010037403 | 1025-1050 | 24 | 9.9 | 20/26 (76%) |
| [HC510346](http://hcv.lanl.gov/components/sequence/HCV/asearch/query_one.comp?se_id=113686&accession=HC510346) | HC510346 | - | - | - | Sequence 72 from Patent WO2010037403 | 1024-1049 | 24 | 9.9 | 20/26 (76%) |
| [HC510347](http://hcv.lanl.gov/components/sequence/HCV/asearch/query_one.comp?se_id=113685&accession=HC510347) | HC510347 | - | - | - | Sequence 73 from Patent WO2010037403 | 1024-1049 | 24 | 9.9 | 20/26 (76%) |
| [HC510358](http://hcv.lanl.gov/components/sequence/HCV/asearch/query_one.comp?se_id=113674&accession=HC510358) | HC510358 | - | - | - | Sequence 84 from Patent WO2010037403 | 1024-1049 | 24 | 9.9 | 20/26 (76%) |
| [HC466804](http://hcv.lanl.gov/components/sequence/HCV/asearch/query_one.comp?se_id=110054&accession=HC466804) | HC466804 | - | - | - | Sequence 18 from Patent WO2010022727 | 1024-1049 | 24 | 9.9 | 20/26 (76%) |
| [HC494118](http://hcv.lanl.gov/components/sequence/HCV/asearch/query_one.comp?se_id=109847&accession=HC494118) | HC494118 |  | - | - | Sequence 751 from Patent WO2009022236 | 6873-6858^1^ | 24 | 10.0 | 15/16 (93%) |
| [HC494172](http://hcv.lanl.gov/components/sequence/HCV/asearch/query_one.comp?se_id=109793&accession=HC494172) | HC494172 | - | - | - | Sequence 805 from Patent WO2009022236 | 982-1007 | 24 | 9.9 | 20/26 (76%) |
| [GU814263](http://hcv.lanl.gov/components/sequence/HCV/asearch/query_one.comp?se_id=113727&accession=GU814263) | S52 | 3a | IT | - | Hepatitis C virus S52 polyprotein gene, complete cds | 1027-1048 | 24 | 9.9 | 20/26 (76%) |
| [HC510279](http://hcv.lanl.gov/components/sequence/HCV/asearch/query_one.comp?se_id=113719&accession=HC510279) | HC510279 | - | - | - | Sequence 5 from Patent WO2010037403 | 1024-1049 | 24 | 9.9 | 20/26 (76%) |
| [HC466795](http://hcv.lanl.gov/components/sequence/HCV/asearch/query_one.comp?se_id=110060&accession=HC466795) | HC466795 | - | - | - | Sequence 9 from Patent WO2010022727 | 1024-1049 | 24 | 9.9 | 20/26 (76%) |
| [FJ515049](http://hcv.lanl.gov/components/sequence/HCV/asearch/query_one.comp?se_id=107097&accession=FJ515049) | D86 | - | TW | 2004 | Hepatitis C virus isolate D86 polyprotein gene, partial cds | 634-659 | 24 | 9.9 | 20/26 (76%) |
| [GQ332546](http://hcv.lanl.gov/components/sequence/HCV/asearch/query_one.comp?se_id=104999&accession=GQ332546) | CYIDU020 | 3a | CY | 2008 | Hepatitis C virus isolate CYIDU020 core-E1 gene, partial cds | 159-184 | 24 | 9.9 | 20/26 (76%) |
| [GQ332549](http://hcv.lanl.gov/components/sequence/HCV/asearch/query_one.comp?se_id=104996&accession=GQ332549) | CYIDU026 | 3a | CY | 2008 | Hepatitis C virus isolate CYIDU026 core-E1 gene, partial cds | 159-184 | 24 | 9.9 | 20/26 (76%) |
| [GQ332550](http://hcv.lanl.gov/components/sequence/HCV/asearch/query_one.comp?se_id=104995&accession=GQ332550) | CYIDU027 | 3a | CY | 2008 | Hepatitis C virus isolate CYIDU027 core-E1 gene, partial cds | 159-184 | 24 | 9.9 | 20/26 (76%) |
| [HC186782](http://hcv.lanl.gov/components/sequence/HCV/asearch/query_one.comp?se_id=104611&accession=HC186782) | HC186782 | - | - | - | Sequence 751 from Patent WO2009130588 | 6873-6858^1^ | 24 | 10.0 | 15/16 (93%) |
| [HC186836](http://hcv.lanl.gov/components/sequence/HCV/asearch/query_one.comp?se_id=104557&accession=HC186836) | HC186836 | - | - | - | Sequence 805 from Patent WO2009130588 | 982-1007 | 24 | 9.9 | 20/26 (76%) |
| [GQ356213](http://hcv.lanl.gov/components/sequence/HCV/asearch/query_one.comp?se_id=104466&accession=GQ356213) | Patient 288 | 3a | GB | 2006 | Hepatitis C virus subtype 3a isolate patient 288 polyprotein gene, partial cds | 684-709 | 24 | 9.9 | 20/26 (76%) |
| [FJ435459](http://hcv.lanl.gov/components/sequence/HCV/asearch/query_one.comp?se_id=93336&accession=FJ435459) | 01AZ048 | 3a | AZ | 2000 | Hepatitis C virus isolate 01AZ048 polyprotein gene, partial cds | 153-178 | 24 | 9.9 | 20/26 (76%) |
| [FJ435464](http://hcv.lanl.gov/components/sequence/HCV/asearch/query_one.comp?se_id=93331&accession=FJ435464) | 01AZ055 | 3a | AZ | 2000 | Hepatitis C virus isolate 01AZ055 polyprotein gene, partial cds | 153-178 | 24 | 9.9 | 20/26 (76%) |
| [FJ435468](http://hcv.lanl.gov/components/sequence/HCV/asearch/query_one.comp?se_id=93327&accession=FJ435468) | 01AZ062 | 3a | AZ | 2000 | Hepatitis C virus isolate 01AZ062 polyprotein gene, partial cds | 153-178 | 24 | 9.9 | 20/26 (76%) |
| [FJ435469](http://hcv.lanl.gov/components/sequence/HCV/asearch/query_one.comp?se_id=93326&accession=FJ435469) | 01AZ065 | 3a | AZ | 2000 | Hepatitis C virus isolate 01AZ065 polyprotein gene, partial cds | 153-178 | 24 | 9.9 | 20/26 (76%) |
| [FJ435473](http://hcv.lanl.gov/components/sequence/HCV/asearch/query_one.comp?se_id=93322&accession=FJ435473) | 01AZ070 | 3a | AZ | 2000 | Hepatitis C virus isolate 01AZ070 polyprotein gene, partial cds | 153-178 | 24 | 9.9 | 20/26 (76%) |
| [FJ435475](http://hcv.lanl.gov/components/sequence/HCV/asearch/query_one.comp?se_id=93320&accession=FJ435475) | 01AZ075 | 3a | AZ | 2000 | Hepatitis C virus isolate 01AZ075 polyprotein gene, partial cds | 153-178 | 24 | 9.9 | 20/26 (76%) |
| [FJ435476](http://hcv.lanl.gov/components/sequence/HCV/asearch/query_one.comp?se_id=93319&accession=FJ435476) | 01AZ076 | 3a | AZ | 2000 | Hepatitis C virus isolate 01AZ076 polyprotein gene, partial cds | 153-178 | 24 | 9.9 | 20/26 (76%) |
| [AY767017](http://hcv.lanl.gov/components/sequence/HCV/asearch/query_one.comp?se_id=52717&accession=AY767017) | 28272-R | 3a | - | - | Hepatitis C virus isolate 28272-R core E1 gene, partial sequence | 93-108 | 24 | 9.9 | 15/16 (93%) |
| [AY767262](http://hcv.lanl.gov/components/sequence/HCV/asearch/query_one.comp?se_id=52472&accession=AY767262) | 32522-R | 3a | - | - | Hepatitis C virus isolate 32522-R core E1 gene, partial sequence | 81-96 | 24 | 9.9 | 15/16 (93%) |

Blast nucleotide homology between homolog E1-E2 HCV fragment (**TT**T**TGTT**TC**CGAAG**C**CAATACATCCA**) generated by PCR and RT-PCR of the studied liver samples and HCV sequences deposited at the site <http://hcv.lanl.gov/content/sequence/BASIC_BLAST/basic_blast.html>.

^1^Reverse complement; CA - Canada, CN - China, NL - Netherlands, DK - Denmark, PT - Portugal, PK - Pakistan, IT - Italy, TW - Taiwan, CY - Cyprus, GB - United Kingdom, AZ - Azerbaijan.
