# Supplementary material for: Endogenous Hepatitis C Virus Homolog Fragments in European Rabbit and Hare Genomes Replicate in Cell Culture
Source: PLoS One. 2012 Nov 19;7(11):e49820. doi: 10.1371/journal.pone.0049820 (PMC3501476; doi:10.1371/journal.pone.0049820)
Supplement: Table S3 — Homolog NS5B HCV genomic fragment present in the European rabbit and Lepus europaeus genomes. (DOC) [file pone.0049820.s006.doc]

**Table S3.** Homolog NS5B HCV genomic fragment present in the European rabbit and *Lepus europaeus* genomes.

| **Accession no.** | **Name** | **Genotype** | **Country** | **Year** | **Description** | **Genome position (nt)** | **Score (bits)** | **E-value** | **Identities** |
| --- | --- | --- | --- | --- | --- | --- | --- | --- | --- |
| [EU246932](http://hcv.lanl.gov/components/sequence/HCV/asearch/query_one.comp?se_id=108254&accession=EU246932) | D88 | 6e | VN | - | Hepatitis C virus strain D88 polyprotein gene, complete cds | 8617-8649 | 54 | 8e-09 | 33/33 (100%) |
| [EU246933](http://hcv.lanl.gov/components/sequence/HCV/asearch/query_one.comp?se_id=108253&accession=EU246933) | D33 | 6l | VN | - | HEpatitis C virus strain D33 polyprotEin gene, complete cds | 8599-8631 | 54 | 8e-09 | 33/33 (100%) |
| [DQ238690](http://hcv.lanl.gov/components/sequence/HCV/asearch/query_one.comp?se_id=31919&accession=DQ238690) | DON77 | 2b | - | - | Hepatitis C virus isolate DON77 NS5B protein (NS5B) gene, partial cds | 66-98 | 54 | 8e-09 | 33/33 (100%) |
| [HQ022879](http://hcv.lanl.gov/components/sequence/HCV/asearch/query_one.comp?se_id=120344&accession=HQ022879) | NT14 | 2b | - | - | Hepatitis C virus subtype 2b isolate NT14 polyprotein gene, partial cds | 287-319 | 51 | 8e-08 | 32/33 (96%) |
| [AB559564](http://hcv.lanl.gov/components/sequence/HCV/asearch/query_one.comp?se_id=112470&accession=AB559564) | acute | 2b | JP | - | Hepatitis C virus HCV gene for hepatitis C virus polyprotein, complete cds | 8678-8710 | 51 | 8e-08 | 32/33 (96%) |
| [FJ859264](http://hcv.lanl.gov/components/sequence/HCV/asearch/query_one.comp?se_id=110936&accession=FJ859264) | CUTH5 | 6f | TH | 2007 | Hepatitis C virus strain CUTH5 polyprotein gene, partial cds | 438-470 | 51 | 8e-08 | 32/33 (96%) |
| [FJ859293](http://hcv.lanl.gov/components/sequence/HCV/asearch/query_one.comp?se_id=110907&accession=FJ859293) | CUTH169 | 6e | TH | 2006 | Hepatitis C virus strain CUTH169 polyprotein gene, partial cds | 408-440 | 51 | 8e-08 | 32/33 (96%) |
| [FJ768772](http://hcv.lanl.gov/components/sequence/HCV/asearch/query_one.comp?se_id=111126&accession=FJ768772) | 03A | 6e | VN | 2008 | Hepatitis C virus isolate 03A polyprotein gene, partial cds | 363-395 | 51 | 8e-08 | 32/33 (96%) |
| [FJ768773](http://hcv.lanl.gov/components/sequence/HCV/asearch/query_one.comp?se_id=111125&accession=FJ768773) | C02 | 6e | VN | 2008 | Hepatitis C virus isolate C02 polyprotein gene, partial cds | 363-395 | 51 | 8e-08 | 32/33 (96%) |
| [FJ768774](http://hcv.lanl.gov/components/sequence/HCV/asearch/query_one.comp?se_id=111124&accession=FJ768774) | C22 | 6e | VN | 2008 | Hepatitis C virus isolate C22 polyprotein gene, partial cds | 363-395 | 51 | 8e-08 | 32/33 (96%) |
| [FJ768799](http://hcv.lanl.gov/components/sequence/HCV/asearch/query_one.comp?se_id=111099&accession=FJ768799) | C15 | 6a | VN | 2008 | Hepatitis C virus isolate C15 polyprotein gene, partial cds | 363-395 | 51 | 8e-08 | 32/33 (96%) |
| [FJ768819](http://hcv.lanl.gov/components/sequence/HCV/asearch/query_one.comp?se_id=111079&accession=FJ768819) | 29A | 1a | VN | 2008 | Hepatitis C virus isolate 29A polyprotein gene, partial cds | 363-395 | 51 | 8e-08 | 32/33 (96%) |
| [HC494101](http://hcv.lanl.gov/components/sequence/HCV/asearch/query_one.comp?se_id=109864&accession=HC494101) | HC494101 | - | - | - | Sequence 734 from Patent WO2009022236 | 8641-8673 | 51 | 8e-08 | 32/33 (96%) |
| [HC494106](http://hcv.lanl.gov/components/sequence/HCV/asearch/query_one.comp?se_id=109859&accession=HC494106) | HC494106 | - | - | - | Sequence 739 from Patent WO2009022236 | 8641-8673 | 51 | 8e-08 | 32/33 (96%) |
| [HC494107](http://hcv.lanl.gov/components/sequence/HCV/asearch/query_one.comp?se_id=109858&accession=HC494107) | HC494107 | - | - | - | Sequence 740 from Patent WO2009022236 | 8641-8673 | 51 | 8e-08 | 32/33 (96%) |
| [HC494145](http://hcv.lanl.gov/components/sequence/HCV/asearch/query_one.comp?se_id=109820&accession=HC494145) | HC494145 | - | - | - | Sequence 778 from Patent WO2009022236 | 8634-8666 | 51 | 8e-08 | 32/33 (96%) |
| [HC494149](http://hcv.lanl.gov/components/sequence/HCV/asearch/query_one.comp?se_id=109816&accession=HC494149) | HC494149 | - | - | - | Sequence 782 from Patent WO2009022236 | 8615-8647 | 51 | 8e-08 | 32/33 (96%) |
| [HC494150](http://hcv.lanl.gov/components/sequence/HCV/asearch/query_one.comp?se_id=109815&accession=HC494150) | HC494150 | - | - | - | Sequence 783 from Patent WO2009022236 | 8624-8656 | 51 | 8e-08 | 32/33 (96%) |
| [DQ363042](http://hcv.lanl.gov/components/sequence/HCV/asearch/query_one.comp?se_id=108729&accession=DQ363042) | BS005 | 1a | BE | 2000 | Hepatitis C virus isolate BS005 NS5B (NS5B) gene, partial cds | 320-352 | 51 | 8e-08 | 32/33 (96%) |
| [DQ363044](http://hcv.lanl.gov/components/sequence/HCV/asearch/query_one.comp?se_id=108727&accession=DQ363044) | DJ004 | 1a | BE | 2000 | Hepatitis C virus isolate DJ004 NS5B (NS5B) gene, partial cds | 320-352 | 51 | 8e-08 | 32/33 (96%) |
| [FJ386799](http://hcv.lanl.gov/components/sequence/HCV/asearch/query_one.comp?se_id=107949&accession=FJ386799) | UN_ELC88_2002.45 | 2b | GB | - | Hepatitis C virus isolate UN_ELC88_2002.45 NS5B gene, partial cds | 66-98 | 51 | 8e-08 | 32/33 (96%) |
| [FJ538021](http://hcv.lanl.gov/components/sequence/HCV/asearch/query_one.comp?se_id=106977&accession=FJ538021) | HCVGR1a6 | 1a | GR | 1996 | Hepatitis C virus isolate HCVGR1a6 polyprotein gene, partial cds | 351-383 | 51 | 8e-08 | 32/33 (96%) |
| [FJ538029](http://hcv.lanl.gov/components/sequence/HCV/asearch/query_one.comp?se_id=106969&accession=FJ538029) | HCVGR1a14 | 1a | GR | 1998 | Hepatitis C virus isolate HCVGR1a14 polyprotein gene, partial cds | 345-377 | 51 | 8e-08 | 32/33 (96%) |
| [FJ538032](http://hcv.lanl.gov/components/sequence/HCV/asearch/query_one.comp?se_id=106966&accession=FJ538032) | HCVGR1a16B | 1a | GR | 1999 | Hepatitis C virus isolate HCVGR1a16B polyprotein gene, partial cds | 351-383 | 51 | 8e-08 | 32/33 (96%) |
| [FJ872340](http://hcv.lanl.gov/components/sequence/HCV/asearch/query_one.comp?se_id=106547&accession=FJ872340) | Ang-5-1a | 1a | FR | - | Hepatitis C virus isolate Ang-5-1a polymerase (NS5B) gene, partial cds | 1011-1043 | 51 | 8e-08 | 32/33 (96%) |
| [FJ872353](http://hcv.lanl.gov/components/sequence/HCV/asearch/query_one.comp?se_id=106534&accession=FJ872353) | TLS8-1a | 1a | FR | - | Hepatitis C virus isolate TLS8-1a polymerase (NS5B) gene, partial cds | 1011-1043 | 51 | 8e-08 | 32/33 (96%) |
| [FJ872364](http://hcv.lanl.gov/components/sequence/HCV/asearch/query_one.comp?se_id=106523&accession=FJ872364) | CF9-5a | 5a | FR | - | Hepatitis C virus isolate CF9-5a polymerase (NS5B) gene, partial cds | 1011-1043 | 51 | 8e-08 | 32/33 (96%) |
| [EU255978](http://hcv.lanl.gov/components/sequence/HCV/asearch/query_one.comp?se_id=105498&accession=EU255978) | HCV-1a/US/BID-V188/1991 | 1a | US | 1991 | Hepatitis C virus subtype 1a isolate HCV-1a/US/BID-V188/1991, complete genome | 8526-8558 | 51 | 8e-08 | 32/33 (96%) |
| [EU155266](http://hcv.lanl.gov/components/sequence/HCV/asearch/query_one.comp?se_id=105796&accession=EU155266) | HCV-1a/US/BID-V390/2006 | 1a | US | 2006 | Hepatitis C virus subtype 1a isolate HCV-1a/US/BID-V390/2006, complete genome | 8481-8513 | 51 | 8e-08 | 32/33 (96%) |
| [EU482862](http://hcv.lanl.gov/components/sequence/HCV/asearch/query_one.comp?se_id=105843&accession=EU482862) | HCV-1a/US/BID-V454/2006 | 1a | US | 2006 | Hepatitis C virus subtype 1a isolate HCV-1a/US/BID-V454/2006, complete genome | 8538-8570 | 51 | 8e-08 | 32/33 (96%) |
| [HC186765](http://hcv.lanl.gov/components/sequence/HCV/asearch/query_one.comp?se_id=104628&accession=HC186765) | HC186765 | - | - | - | Sequence 734 from Patent WO2009130588 | 8641-8673 | 51 | 8e-08 | 32/33 (96%) |
| [HC186770](http://hcv.lanl.gov/components/sequence/HCV/asearch/query_one.comp?se_id=104623&accession=HC186770) | HC186770 | - | - | - | Sequence 739 from Patent WO2009130588 | 8641-8673 | 51 | 8e-08 | 32/33 (96%) |
| [HC186771](http://hcv.lanl.gov/components/sequence/HCV/asearch/query_one.comp?se_id=104622&accession=HC186771) | HC186771 | - | - | - | Sequence 740 from Patent WO2009130588 | 8641-8673 | 51 | 8e-08 | 32/33 (96%) |
| [FJ463015](http://hcv.lanl.gov/components/sequence/HCV/asearch/query_one.comp?se_id=103971&accession=FJ463015) | DL308 | 6u | CN | 2008 | Hepatitis C virus isolate DL308 nonfunctional RNA-dependent RNA polymerase mRNA, partial sequence | 275-307 | 51 | 8e-08 | 32/33 (96%) |
| [EU529680](http://hcv.lanl.gov/components/sequence/HCV/asearch/query_one.comp?se_id=105679&accession=EU529680) | HCV-1a/US/BID-V80/2002 | 1a | US | 2002 | Hepatitis C virus subtype 1a isolate HCV-1a/US/BID-V80/2002, complete genome | 8549-8581 | 51 | 8e-08 | 32/33 (96%) |
| [EU256104](http://hcv.lanl.gov/components/sequence/HCV/asearch/query_one.comp?se_id=105602&accession=EU256104) | HCV-1a/US/BID-V430/2005 | 1a | US | 2005 | Hepatitis C virus subtype 1a isolate HCV-1a/US/BID-V430/2005, complete genome | 8541-8573 | 51 | 8e-08 | 32/33 (96%) |
| [HC186809](http://hcv.lanl.gov/components/sequence/HCV/asearch/query_one.comp?se_id=104584&accession=HC186809) | HC186809 | - | - | - | Sequence 778 from Patent WO2009130588 | 8634-8666 | 51 | 8e-08 | 32/33 (96%) |
| [HC186813](http://hcv.lanl.gov/components/sequence/HCV/asearch/query_one.comp?se_id=104580&accession=HC186813) | HC186813 | - | - | - | Sequence 782 from Patent WO2009130588 | 8615-8647 | 51 | 8e-08 | 32/33 (96%) |
| [HC186814](http://hcv.lanl.gov/components/sequence/HCV/asearch/query_one.comp?se_id=104579&accession=HC186814) | HC186814 | - | - | - | Sequence 783 from Patent WO2009130588 | 8624-8656 | 51 | 8e-08 | 32/33 (96%) |
| [FJ024132](http://hcv.lanl.gov/components/sequence/HCV/asearch/query_one.comp?se_id=100400&accession=FJ024132) | 18783 | 1a | NL | 1992 | Hepatitis C virus isolate 18783 NS5B protein gene, partial cds | 66-98 | 51 | 8e-08 | 32/33 (96%) |
| [FJ024133](http://hcv.lanl.gov/components/sequence/HCV/asearch/query_one.comp?se_id=100399&accession=FJ024133) | 18783 | 1a | NL | 1993 | Hepatitis C virus isolate 18783 NS5B protein gene, partial cds | 66-98 | 51 | 8e-08 | 32/33 (96%) |
| [FJ024134](http://hcv.lanl.gov/components/sequence/HCV/asearch/query_one.comp?se_id=100398&accession=FJ024134) | 18783 | 1a | NL | 1994 | Hepatitis C virus isolate 18783 NS5B protein gene, partial cds | 66-98 | 51 | 8e-08 | 32/33 (96%) |
| [FJ024135](http://hcv.lanl.gov/components/sequence/HCV/asearch/query_one.comp?se_id=100397&accession=FJ024135) | 18783 | 1a | NL | 1997 | Hepatitis C virus isolate 18783 NS5B protein gene, partial cds | 66-98 | 51 | 8e-08 | 32/33 (96%) |
| [FJ024136](http://hcv.lanl.gov/components/sequence/HCV/asearch/query_one.comp?se_id=100396&accession=FJ024136) | 18783 | 1a | NL | 2001 | Hepatitis C virus isolate 18783 NS5B protein gene, partial cds | 66-98 | 51 | 8e-08 | 32/33 (96%) |
| [FJ024137](http://hcv.lanl.gov/components/sequence/HCV/asearch/query_one.comp?se_id=100395&accession=FJ024137) | 18783 | 1a | NL | 2005 | Hepatitis C virus isolate 18783 NS5B protein gene, partial cds | 66-98 | 51 | 8e-08 | 32/33 (96%) |
| [FJ024155](http://hcv.lanl.gov/components/sequence/HCV/asearch/query_one.comp?se_id=100377&accession=FJ024155) | 19713 | 1a | NL | 2005 | Hepatitis C virus isolate 19713 NS5B protein gene, partial cds | 66-98 | 51 | 8e-08 | 32/33 (96%) |
| [EU684627](http://hcv.lanl.gov/components/sequence/HCV/asearch/query_one.comp?se_id=91300&accession=EU684627) | 56 | 1a | CY | 2005 | Hepatitis C virus strain CYHCV056 NS5B gene, partial cds | 336-368 | 51 | 8e-08 | 32/33 (96%) |
| [EU408326](http://hcv.lanl.gov/components/sequence/HCV/asearch/query_one.comp?se_id=101602&accession=EU408326) | 537798 | 6e | US | - | Hepatitis C virus isolate 537798 polyprotein precursor, gene, complete cds | 8642-8674 | 49 | 2e-07 | 31/33 (93%) |

Blast nucleotide homology between homolog RdRp-NS5B HCV fragment (**AGCTTTCACGGAGGCTATGACCAGGTACTCAGC**) generated by PCR and RT-PCR of the studied liver samples and HCV sequences deposited at the site <http://hcv.lanl.gov/content/sequence/BASIC_BLAST/basic_blast.html>.. VN - Viet Nam, JP - Japan, TH - Thailand, BE - Belgium, GB - United Kingdom, GR - Greece, FR - France, US - USA, CN - China, NL - Netherlands, CY - Cyprus.
