# Supplementary material for: Endogenous Hepatitis C Virus Homolog Fragments in European Rabbit and Hare Genomes Replicate in Cell Culture
Source: PLoS One. 2012 Nov 19;7(11):e49820. doi: 10.1371/journal.pone.0049820 (PMC3501476; doi:10.1371/journal.pone.0049820)
Supplement: Table S4 — Blastn between HCV 1b, O. cuniclus and H. sapiens. (DOC) [file pone.0049820.s007.doc]

**Table S4.** Blastn between HCV 1b, *O. cuniclus* and *H. sapiens*.

| **HCV region** | **Start** | **End** | **Specie** | **Scaffold, chromosome or accession no.** | **Start** | **End** | **Score** | **E-value** | **% Identities** | **Length** |
| --- | --- | --- | --- | --- | --- | --- | --- | --- | --- | --- |
| Complete sequence (1-9413)  9413bp | 784 | 812 | *O. cuniculus* | Scaffold GL018847 | 669,923 | 669,950 | 21 | 0.11 | 93.1 | 29 |
|  | 1,370 | 1,385 | *O. cuniculus* | Scaffold GL018847 | 489,816 | 489,831 | 16 | 0.11 | 100 | 16 |
|  | 2,658 | 2,672 | *O. cuniculus* | Scaffold GL018847 | 136,289 | 136,303 | 15 | 7.2 | 100 | 15 |
|  | 3,785 | 3,799 | *O. cuniculus* | Scaffold GL018847 | 178,090 | 178,104 | 15 | 7.2 | 100 | 15 |
|  | 3,788 | 3,806 | *O. cuniculus* | Scaffold GL018847 | 353,402 | 353,420 | 19 | 0.11 | 100 | 19 |
|  | 4,420 | 4,434 | *O. cuniculus* | Scaffold GL018847 | 86,718 | 86,732 | 15 | 0.11 | 100 | 15 |
|  | 5,558 | 5,573 | *O. cuniculus* | Scaffold GL018847 | 59,212 | 59,227 | 16 | 0.11 | 100 | 16 |
|  | 6,529 | 6,545 | *O. cuniculus* | Scaffold GL018847 | 18,455 | 18,471 | 17 | 0.11 | 100 | 17 |
|  | 6,663 | 6,685 | *O. cuniculus* | Scaffold GL018847 | 243,654 | 243,676 | 16 | 0.82 | 91.67 | 24 |
|  | 443 | 457 | *O. cuniculus* | Scaffold GL018930 | 199,306 | 199,320 | 15 | 1.7 | 100 | 15 |
|  | 443 | 457 | *O. cuniculus* | Scaffold GL018930 | 199,344 | 199,358 | 15 | 1.7 | 100 | 15 |
|  | 1,518 | 1,541 | *O. cuniculus* | Scaffold GL018930 | 242,629 | 242,652 | 24 | 1.7 | 100 | 24 |
|  | 3,792 | 3,806 | *O. cuniculus* | Scaffold GL018930 | 299,337 | 299,351 | 15 | 5.3 | 100 | 15 |
|  | 5,896 | 5,911 | *O. cuniculus* | Scaffold GL018930 | 295,360 | 295,375 | 16 | 1.7 | 100 | 16 |
|  | --- | --- | *H. sapiens* | --- | --- | --- | --- | --- | --- | --- |
| 5`UTR (1-329)  329bp | 94 | 118 | *O. cuniculus* | Scaffold GL018725 | 2,241,470 | 2,241,494 | 21 | 0.41 | 96.00 | 25 |
|  | 103 | 121 | *O. cuniculus* | Scaffold GL018915 | 161,662 | 161,680 | 19 | 2 | 100 | 19 |
|  | 183 | 197 | *O. cuniculus* | Scaffold GL018915 | 340,159 | 340,173 | 182 | 197 | 100 | 15 |
|  | --- | --- | *H. sapiens* | --- | --- | --- | --- | --- | --- | --- |
| Core (330-902)  573bp | 242 | 257 | *O. cuniculus* | Chr 3 | 154,378,789 | 154,378,804 | 16 | 6.5 | 100 | 16 |
|  | 266 | 283 | *O. cuniculus* | Chr 3 | 144,272,702 | 144,272,719 | 18 | 6.5 | 100 | 18 |
|  | 439 | 457 | *O. cuniculus* | Chr 3 | 143,785,979 | 143,785,997 | 19 | 6.5 | 100 | 19 |
|  | 516 | 535 | *O. cuniculus* | Chr 3 | 141,539,808 | 141,539,827 | 20 | 6.5 | 100 | 20 |
|  | 540 | 555 | *O. cuniculus* | Chr 3 | 139,566,970 | 139,566,985 | 16 | 6.5 | 100 | 16 |
|  | 274 | 288 | *O. cuniculus* | Scaffold GL018734 | 528,846 | 528,860 | 15 | 3.7 | 100 | 15 |
|  | 321 | 337 | *O. cuniculus* | Scaffold GL018734 | 659,311 | 659,327 | 17 | 3.7 | 100 | 17 |
|  | 443 | 457 | *O. cuniculus* | Scaffold GL018734 | 951,609 | 951,623 | 15 | 3.7 | 100 | 15 |
|  | 519 | 536 | *O. cuniculus* | Scaffold GL018734 | 1,527,059 | 1,527,076 | 18 | 3.7 | 100 | 18 |
|  | 221 | 240 | *O. cuniculus* | Scaffold GL018801 | 779,733 | 779,752 | 20 | 2.9 | 100 | 20 |
|  | 455 | 483 | *O. cuniculus* | Scaffold GL018847 | 669,923 | 669,950 | 21 | 0.73 | 93.10 | 29 |
|  | 288 | 306 | *O. cuniculus* | Scaffold GL018928 | 367,988 | 368,006 | 19 | 6.3 | 100 | 19 |
|  | 515 | 529 | *O. cuniculus* | Scaffold GL018928 | 230,806 | 230,820 | 15 | 6.3 | 100 | 15 |
|  | --- | --- | *H. sapiens* | --- | --- | --- | --- | --- | --- | --- |
| E1 (903-1478) 576bp | 284 | 300 | *O. cuniculus* | Chr 7 | 162,457,019 | 162,457,035 | 17 | 9.4 | 100 | 17 |
|  | 469 | 488 | *O. cuniculus* | Chr 7 | 159,709,689 | 159,709,708 | 20 | 9.4 | 100 | 20 |
|  | 504 | 518 | *O. cuniculus* | Chr 7 | 146,722,757 | 146,722,771 | 15 | 9.4 | 100 | 15 |
|  | 535 | 554 | *O. cuniculus* | Chr 7 | 137,663,087 | 137,663,106 | 20 | 9.4 | 100 | 20 |
|  | 385 | 399 | *O. cuniculus* | Scaffold GL018937 | 102,913 | 102,927 | 15 | 5.9 | 100 | 15 |
|  | 464 | 482 | *O. cuniculus* | Scaffold GL018937 | 25,801 | 25,819 | 19 | 5.9 | 100 | 19 |
|  | --- | --- | *H. sapiens* | --- | --- | --- | --- | --- | --- | --- |
| E2 (1479-2567) 1089bp | 40 | 63 | *O. cuniculus* | Scaffold GL018930 | 242,629 | 242,652 | 24 | 0.023 | 100 | 24 |
|  | 245 | 262 | *O. cuniculus* | Scaffold GL018730 | 714,115 | 714,132 | 18 | 8.2 | 100 | 18 |
|  | 798 | 819 | *O. cuniculus* | Scaffold GL018730 | 292,158 | 292,178 | 18 | 8.2 | 95.35 | 22 |
|  | 643 | 657 | *O. cuniculus* | Scaffold GL018970 | 123,216 | 123,230 | 15 | 1.6 | 100 | 15 |
|  | 983 | 1,003 | *O. cuniculus* | Scaffold GL018970 | 233,113 | 233,133 | 21 | 1.6 | 100 | 21 |
|  | 983 | 1,003 | *O. cuniculus* | AccessionAAGW02079307 | 85,276 | 85,296 | 21 | 1.4 | 100 | 21 |
|  | 983 | 1,003 | *O. cuniculus* | AccessionAAGW02082840 | 4,521 | 4,541 | 21 | 1.4 | 100 | 21 |
|  | --- | --- | *H. sapiens* | --- | --- | --- | --- | --- | --- | --- |
| P7 (2568-2756) 189bp | 148 | 165 | *O. cuniculus* | Chr 11 | 70,530,554 | 70,530,571 | 18 | 5.6 | 100 | 18 |
|  | 99 | 114 | *O. cuniculus* | Chr 11 | 84,620,409 | 84,620,424 | 16 | 5.6 | 100 | 16 |
|  | 34 | 59 | *O. cuniculus* | Chr 11 | 85,502,619 | 85,502,642 | 18 | 5.6 | 92.31 | 26 |
|  | 50 | 69 | *O. cuniculus* | Chr 19 | 50,057,114 | 50,057,133 | 20 | 8.9 | 100 | 20 |
|  | 140 | 154 | *O. cuniculus* | Chr 19 | 53,250,209 | 53,250,223 | 15 | 8.9 | 100 | 15 |
|  | 100 | 118 | *O. cuniculus* | Scaffold GL018883 | 389,014 | 389,032 | 19 | 3.5 | 100 | 19 |
|  | --- | --- | *H. sapiens* | --- | --- | --- | --- | --- | --- | --- |
| NS2 (2757-3407) 651bp | --- | --- | *O. cuniculus* | --- | --- | --- | --- | --- | --- | --- |
|  | --- | --- | *H. sapiens* | --- | --- | --- | --- | --- | --- | --- |
| NS3 (3408-5300) 1893bp | 1,099 | 1,117 | *O. cuniculus* | Scaffold GL018742 | 2,194,270 | 2,194,288 | 19 | 5.3 | 100 | 19 |
|  | 1,571 | 1,588 | *O. cuniculus* | Scaffold GL018742 | 1,351,265 | 1,351,282 | 18 | 5.3 | 100 | 18 |
|  | 1,207 | 1,226 | *O. cuniculus* | Scaffold GL018831 | 681,448 | 681,467 | 20 | 9.8 | 100 | 20 |
|  | 466 | 485 | *O. cuniculus* | Scaffold GL019027 | 206,549 | 206,568 | 20 | 9.8 | 100 | 20 |
|  | --- | --- | *H. sapiens* | --- | --- | --- | --- | --- | --- | --- |
| NS4A (5301-5462) 162bp | 67 | 83 | *O. cuniculus* | Chr 20 | 6,168,581 | 6,168,597 | 17 | 9.1 | 100 | 17 |
|  | 96 | 111 | *O. cuniculus* | Chr 20 | 28,075,207 | 28,075,222 | 16 | 9.1 | 100 | 16 |
|  | 125 | 141 | *O. cuniculus* | Chr 20 | 32,857,558 | 32,857,574 | 17 | 9.1 | 100 | 17 |
|  | --- | --- | *H. sapiens* | --- | --- | --- | --- | --- | --- | --- |
| NS4B (5463-6245) 783bp | 152 | 176 | *O. cuniculus* | Scaffold GL018705 | 4,726,064 | 4,726,087 | 21 | 2.4 | 96 | 25 |
|  | 199 | 214 | *O. cuniculus* | Scaffold GL018705 | 1,558,979 | 1,558,994 | 16 | 2.4 | 100 | 16 |
|  | 199 | 214 | *O. cuniculus* | Scaffold GL018705 | 1,704,924 | 1,704,939 | 16 | 2.4 | 100 | 16 |
|  | 199 | 214 | *O. cuniculus* | Scaffold GL018705 | 1,747,958 | 1,747,973 | 16 | 2.4 | 100 | 16 |
|  | 199 | 214 | *O. cuniculus* | Scaffold GL018705 | 2,713,008 | 2,713,023 | 16 | 2.4 | 100 | 16 |
|  | 199 | 214 | *O. cuniculus* | Scaffold GL018705 | 2,727,760 | 2,727,775 | 16 | 2.4 | 100 | 16 |
|  | 199 | 214 | *O. cuniculus* | Scaffold GL018705 | 2,777,826 | 2,777,841 | 16 | 2.4 | 100 | 16 |
|  | 199 | 214 | *O. cuniculus* | Scaffold GL018705 | 2,919,349 | 2,919,364 | 16 | 2.4 | 100 | 16 |
|  | 199 | 214 | *O. cuniculus* | Scaffold GL018705 | 3,054,107 | 3,054,122 | 16 | 2.4 | 100 | 16 |
|  | 199 | 214 | *O. cuniculus* | Scaffold GL018705 | 3,252,234 | 3,252,249 | 16 | 2.4 | 100 | 16 |
|  | 199 | 214 | *O. cuniculus* | Scaffold GL018705 | 3,484,288 | 3,484,303 | 16 | 2.4 | 100 | 16 |
|  | 199 | 214 | *O. cuniculus* | Scaffold GL018705 | 3,506,363 | 3,506,378 | 16 | 2.4 | 100 | 16 |
|  | 199 | 214 | *O. cuniculus* | Scaffold GL018705 | 3,643,318 | 3,643,333 | 16 | 2.4 | 100 | 16 |
|  | 199 | 214 | *O. cuniculus* | Scaffold GL018705 | 3,819,239 | 3,819,254 | 16 | 2.4 | 100 | 16 |
|  | 199 | 214 | *O. cuniculus* | Scaffold GL018705 | 4,177,866 | 4,177,881 | 16 | 2.4 | 100 | 16 |
|  | 199 | 214 | *O. cuniculus* | Scaffold GL018705 | 4,201,059 | 4,201,074 | 16 | 2.4 | 100 | 16 |
|  | 261 | 276 | *O. cuniculus* | Scaffold GL018712 | 34,491 | 34,506 | 16 | 3.6 | 100 | 16 |
|  | 199 | 218 | *O. cuniculus* | Scaffold GL018712 | 1,229,482 | 1,229,501 | 20 | 3.6 | 100 | 20 |
|  | 89 | 104 | *O. cuniculus* | Scaffold GL018712 | 2,956,419 | 2,956,434 | 16 | 3.6 | 100 | 16 |
|  | 3 | 17 | *O. cuniculus* | Scaffold GL018712 | 4,329,670 | 4,329,684 | 15 | 3.6 | 100 | 15 |
|  | 585 | 602 | *O. cuniculus* | Scaffold GL018734 | 286,939 | 286,956 | 18 | 4.3 | 100 | 18 |
|  | 765 | 782 | *O. cuniculus* | Scaffold GL018734 | 1,033,368 | 1,033,385 | 18 | 4.3 | 100 | 18 |
|  | 139 | 153 | *O. cuniculus* | Scaffold GL018789 | 251,025 | 251,039 | 15 | 1.1 | 100 | 15 |
|  | 199 | 214 | *O. cuniculus* | Scaffold GL018789 | 274,310 | 274,325 | 16 | 1.1 | 100 | 16 |
|  | 335 | 349 | *O. cuniculus* | Scaffold GL018789 | 279,912 | 279,926 | 15 | 1.1 | 100 | 15 |
|  | 437 | 451 | *O. cuniculus* | Scaffold GL018789 | 280,010 | 280,024 | 15 | 1.1 | 100 | 15 |
|  | 647 | 664 | *O. cuniculus* | Scaffold GL018789 | 649,287 | 649,304 | 18 | 1.1 | 100 | 18 |
|  | 199 | 218 | *O. cuniculus* | Scaffold GL018790 | 394,481 | 394,500 | 20 | 7.4 | 100 | 20 |
|  | 3 | 17 | *O. cuniculus* | Scaffold GL018790 | 900,012 | 900,026 | 15 | 7.4 | 100 | 15 |
|  | 199 | 218 | *O. cuniculus* | Scaffold GL018901 | 212,128 | 212,147 | 20 | 4 | 100 | 20 |
|  | 376 | 390 | *O. cuniculus* | Scaffold GL018901 | 508,410 | 508,424 | 15 | 4 | 100 | 15 |
|  | --- | --- | *H. sapiens* | --- | --- | --- | --- | --- | --- | --- |
| NS5A (6246-7586) 1341bp | 57 | 71 | *O. cuniculus* | Scaffold GL018704 | 821,505 | 821,519 | 15 | 7.9 | 100 | 15 |
|  | 482 | 496 | *O. cuniculus* | Scaffold GL018704 | 1,536,602 | 1,536,616 | 15 | 7.9 | 100 | 15 |
|  | 335 | 349 | *O. cuniculus* | Scaffold GL018704 | 4,161,678 | 4,161,692 | 15 | 7.9 | 100 | 15 |
|  | 649 | 674 | *O. cuniculus* | Scaffold GL018704 | 5,516,931 | 5,516,957 | 23 | 7.9 | 96.3 | 27 |
|  | 1,255 | 1,284 | *O. cuniculus* | Scaffold GL018847 | 472,017 | 472,045 | 22 | 0.45 | 93.33 | 30 |
|  | 546 | 567 | *O. cuniculus* | Scaffold GL019488 | 15,125 | 15,145 | 18 | 1.5 | 95.45 | 22 |
|  | 785 | 801 | *O. cuniculus* | Scaffold GL019488 | 37,344 | 37,360 | 17 | 1.5 | 100 | 17 |
|  | --- | --- | *H. sapiens* | --- | --- | --- | --- | --- | --- | --- |
| NS5B (7587-9359) 1773bp | --- | --- | *O. cuniculus* | --- | --- | --- | --- | --- | --- | --- |
|  | --- | --- | *H. sapiens* | --- | --- | --- | --- | --- | --- | --- |
| 3`UTR (9363-9413) 51bp | 19 | 35 | *O. cuniculus* | Chr 4 | 53,293,483 | 53,293,499 | 17 | 6 | 100 | 17 |
|  | 17 | 35 | *O. cuniculus* | Chr 4 | 67,935,373 | 53,293,499 | 17 | 6 | 100 | 17 |
|  | 19 | 35 | *O. cuniculus* | Chr 9 | 91,599,649 | 91,599,665 | 17 | 6 | 100 | 17 |
|  | 5 | 22 | *H. sapiens* | Chr 6 | 69,855,721 | 69,855,738 | 18 | 9.8 | 100 | 18 |
|  | 5 | 22 | *H. sapiens* | Chr 13 | 86,509,933 | 86,509,950 | 18 | 9.8 | 100 | 18 |

Nucleotide homology between HCV 1b (ID - D90208, HCV database) *O. cuniculus* and *H.sapiens* genomes deposited at the ensembl site (http://www.ensembl.org/Multi/blastview), complete 5'UTR, complete CDS (Contains: C, E1, E2, P7, NS2, NS3, NS4A, NS4B, NS5A, NS5B) and partial 3'UTR. Species Blastn was performed with a selected DNAdatabase and search sensitivity near- exact matches’ options.
