# Supplementary material for: Endogenous Hepatitis C Virus Homolog Fragments in European Rabbit and Hare Genomes Replicate in Cell Culture
Source: PLoS One. 2012 Nov 19;7(11):e49820. doi: 10.1371/journal.pone.0049820 (PMC3501476; doi:10.1371/journal.pone.0049820)
Supplement: Table S5 — All HCV homolog proteins identified by MALDI-TOF/TOF-MS/MS analysis from the European rabbit and Lepus europaeus liver homogenates. (DOC) [file pone.0049820.s008.doc]

**Table S5**. All HCV homolog proteins identified by MALDI-TOF/TOF-MS/MS analysis from the European rabbit and *Lepus europaeus* liver homogenates.

| **Animal** | **Accession no..^Ϯ^ (genotype)** | **Protein name** | **Best peptide sequence** | **Genome position (aa)** | **Calculated mass (Da)** | **Observed mass (Da)** | **Match error (Da)** | **Total ion score C I %** | **E-value** |
| --- | --- | --- | --- | --- | --- | --- | --- | --- | --- |
| DR | POLG_HCVJA (1b) | Core | STNPKPQR | 2-9 | 927.50 | 927.48 | -0.02 | 99 | 0.39 |
| DR | POLG_HCVJA (1b) | Core | SQPRGRR | 56-62 | 856.49 | 856.47 | -0.02 | 99 | 0.23 |
| DR | POLG_HCVJA (1b) | Core | TWAQPGYPWPLYGNEGMGWAGWLLSPR | 75-101 | 3090.47 | 3090.54 | 0.07 | 99 | 0.3 |
| DR | POLG_HCVJA (1b) | Envelope glycoprotein E1 | NSSIPTTTIRR | 250-260 | 1245.69 | 1245.65 | -0.04 | 99 | 0.022 |
| DR | POLG_HCVJA (1b) | Envelope glycoprotein E2 | VASSTQSLVSWLSQGPSQK | 392-410 | 1990.02 | 1989.95 | -0.07 | 98 | 0.046 |
| DR | POLG_HCVVA (2K) | Envelope glycoprotein E2 | SIEEFR | 461-466 | 780.39 | 780.42 | 0.03 | 71 | 0.58 |
| DR | POLG_HCVBK (1b) | Protease NS2-3 | KVAGGHYVQMAFMK** | 927-940 | 1598.78 | 1598.74 | -0.04 | 95 | 0.047 |
| DR | POLG_HCVJA (1b) | Serine protease NS3 | GPITQMYTNVDQDLVGWPAPPGAR | 1095-1118 | 2583.27 | 2583.32 | 0.06 | 81 | 0.6 |
| DR | POLG_HCVJ1 (1b) | Serine protease NS3 | AVDFIPVESLETTMR | 1192-1206 | 1707.86 | 1707.79 | -0.07 | 87 | 0.049 |
| DR | POLG_HCVJA (1b) | Non-structural protein 5A | DVWDWICTVLSDFKTWLQSKLLPR* | 1979-2002 | 3006.55 | 3006.65 | 0.09 | 81 | 0.013 |
| DR | POLG_HCVT5 (6b) | Non-structural protein 5A | IPGIPFISCQAGYR* | 2008-2021 | 1578.81 | 1578.80 | -0.01 | 89 | 0.15 |
| DR | POLG_HCVK3 (3a) | Non-structural protein 5A | NGSMRLAGPR** | 2047-2056 | 1074.55 | 1074.53 | -0.02 | 74 | 0.92 |
| DR | POLG_HCVSA (5a) | Non-structural protein 5A | GSPPSLASSSASQLSAPSLK | 2194-2213 | 1872.97 | 1871.89 | -0.08 | 90 | 0.087 |
| DR | POLG_HCVJA (1b) | RdRp-NS5B | VEFLVNTWK | 2620-2628 | 1135.61 | 1135.62 | 0.01 | 81 | 0.15 |
| DR | POLG_HCVSA (5a) | RdRp-NS5B | AAIRSLTQR | 2674-2682 | 1015.6 | 1015.58 | -0.02 | 99 | 0.1 |
| DR | POLG_HCVJA (1b) | RdRp-NS5B | AFTEAMTR | 2757-2764 | 926.44 | 926.43 | -0.01 | 81 | 1.1 |
| WR | POLG_HCVNZ (3a) | Core | SQPRGRR | 56-62 | 856.49 | 856.52 | 0.04 | 22 | 1.1 |
| WR | POLG_HCV6A (6a) | Serine protease NS3 | CDELAGKLKSLGLNAVAFYR* | 1405-1424 | 2225.17 | 2225.13 | -0,04 | 87 | 0.15 |
| WR | POLG_HCVJ8 (2b) | Serine protease NS3 | GRLGVYR | 1498-1504 | 820.48 | 820.44 | -0.04 | 57 | 0.05 |
| WR | POLG_HCVJF (2a) | Serine protease NS3 | AKAPPPSWDAMWKCLAR* | 1601-1617 | 2000.98 | 2001.02 | 0.04 | 93 | 0.016 |
| WR | POLG_HCVVO (6K) | Non-structural protein 5A | NGSMRISGSR | 2043-2052 | 1064.53 | 1064.57 | 0.04 | 22 | 0.05 |
| WR | POLG_HCVVN (6d) | Non-structural protein 5A | IVGPKMCSNVWNNR* | 2044-2057 | 1690.82 | 1690.83 | 0.01 | 87 | 0.2 |
| WR | POLG_HCVCO (1b) | Non-structural protein 5A | VGDFHYVTGMTTDNVK** | 2096-2111 | 1799.83 | 1799.89 | 0.06 | 74 | 0.5 |
| WR | POLG_HCVCO (1b) | Non-structural protein 5A | GSPPSLASSSASQLSAPSLK | 2193-2212 | 1871.97 | 1871.89 | -0.08 | 74 | 0.53 |
| WR | POLG_HCVJ6 (2a) | Non-structural protein 5A | SDLEPSIPSEYMLPKKR | 2264-2280 | 1990.03 | 1989.98 | -0.05 | 22 | 0.1 |
| WR | POLG_HCV6A (6a) | RdRp-NS5B | SASLRQK | 2472-2478 | 789.46 | 789.46 | 0.00 | 87 | 0.13 |
| WR | POLG_HCVJ8 (2b) | RdRp-NS5B | LLTVEEACALTPPHSAK* | 2524-2540 | 1836.95 | 1836.90 | -0,05 | 57 | 0.15 |
| WR | POLG_HCV6A (6a) | RdRp-NS5B | MALYDVTR** | 2601-2608 | 984.48 | 984.49 | 0.01 | 87 | 0.2 |
| Hare | POLG_HCVJP (2b) | Core | GSRPTWGPSDPRHR | 102-115 | 1605.79 | 1605.84 | 0.04 | 53 | 0.13 |
| Hare | POLG_HCVJP (2b) | Envelope glycoprotein E2 | LWHYPCTVNFTIFKVR* | 619-634 | 2081.08 | 2081.04 | -0.04 | 53 | 0.65 |
| Hare | POLG_HCVJK (3K) | Protease NS2-3 | LGKEVLLGPADDYR | 1011-1024 | 1545.83 | 1545.76 | -0.07 | 81 | 0.067 |
| Hare | POLG_HCVK3 (3a) | Non-structural protein 5A | NGSMRLAGPR** | 2047-2056 | 1074.55 | 1074.50 | -0.05 | 67 | 0.92 |
| Hare | POLG_HCVJP (2b) | RdRp-NS5B | AASKVSAR | 2516-2523 | 789.46 | 789.46 | 0.00 | 53 | 0.65 |
| Hare | POLG_HCVT5 (6b) | RdRp-NS5B | DVRSHTSK | 2535-2542 | 929.48 | 929.52 | 0.04 | 82 | 0.09 |

DR – Domestic rabbit *(Oryctolagus cuniculus)*, WR – Wild rabbit *(Oryctolagus cuniculus.),* Hare *(Lepus europaeu*), RdRp-NS5B - RNA-directed RNA polymerase –Non-structural protein 5B, Ϯ – SwissProt accession number,*Modification - Carbamidomethyl (C), ** Modification - Oxidation (M).
