# Supplementary material for: Endogenous Hepatitis C Virus Homolog Fragments in European Rabbit and Hare Genomes Replicate in Cell Culture
Source: PLoS One. 2012 Nov 19;7(11):e49820. doi: 10.1371/journal.pone.0049820 (PMC3501476; doi:10.1371/journal.pone.0049820)
Supplement: Table S6 — Blastn between HCV specific primers and O. cuniclus. (DOC) [file pone.0049820.s009.doc]

**Table S6.** Blastn between HCV specific primers and *O. cuniclus*.

| **HCV specific primer** | **Start** | **End** | **Specie** | **Scaffold or chromosome** | **Start** | **End** | **Score** | **E-value** | **% Identities** | **Length** |
| --- | --- | --- | --- | --- | --- | --- | --- | --- | --- | --- |
| 256 (Core PCR I) | 5 | 19 | *O. cuniculus* | Chr 1 | 111,447,230 | 111,447,244 | 15 | 9.2 | 100 | 15 |
|  | 5 | 19 | *O. cuniculus* | Chr 7 | 120,353,256 | 120,353,270 | 15 | 9.3 | 100 | 15 |
|  | 6 | 20 | *O. cuniculus* | Chr 13 | 43,700,904 | 43,700,918 | 15 | 9.4 | 100 | 15 |
|  | 6 | 20 | *O. cuniculus* | Chr 16 | 29,734,194 | 29,734,208 | 15 | 9.7 | 100 | 15 |
|  | 6 | 20 | *O. cuniculus* | Chr 16 | 30,084,642 | 30,084,656 | 15 | 9.7 | 100 | 15 |
|  | 6 | 20 | *O. cuniculus* | Chr 16 | 30,136,175 | 30,136,189 | 15 | 9.7 | 100 | 15 |
|  | 2 | 16 | *O. cuniculus* | Chr 5 | 37,677,771 | 37,677,785 | 15 | 9.9 | 100 | 15 |
| 186 (Core PCR I) | 2 | 19 | *O. cuniculus* | Chr 9 | 20,683,441 | 20,683,458 | 18 | 0.082 | 100 | 18 |
|  | 4 | 19 | *O. cuniculus* | Chr 7 | 160,657,698 | 160,657,713 | 16 | 1.3 | 100 | 16 |
|  | 2 | 17 | *O. cuniculus* | Chr 12 | 130,454,587 | 130,454,602 | 16 | 1.3 | 100 | 16 |
|  | 2 | 17 | *O. cuniculus* | Chr 12 | 149,542,367 | 149,542,382 | 16 | 1.3 | 100 | 16 |
|  | 4 | 19 | *O. cuniculus* | Chr 13 | 114,051,832 | 114,051,847 | 16 | 1.3 | 100 | 16 |
|  | 2 | 17 | *O. cuniculus* | Chr 16 | 13,968,682 | 13,968,697 | 16 | 1.3 | 100 | 16 |
|  | 2 | 16 | *O. cuniculus* | Chr 7 | 76,693,588 | 76,693,602 | 15 | 9.3 | 100 | 15 |
|  | 6 | 20 | *O. cuniculus* | Chr 13 | 119,239,226 | 119,239,240 | 15 | 9.4 | 100 | 15 |
|  | 3 | 17 | *O. cuniculus* | Chr 1 | 98,480,676 | 98,480,690 | 15 | 9.2 | 100 | 15 |
|  | 6 | 20 | *O. cuniculus* | Chr 2 | 140,388,225 | 140,388,239 | 15 | 9.3 | 100 | 15 |
|  | 4 | 18 | *O. cuniculus* | Chr 2 | 47,564,932 | 47,564,946 | 15 | 9.3 | 100 | 15 |
|  | 5 | 19 | *O. cuniculus* | Chr 2 | 56,095,890 | 56,095,904 | 15 | 9.3 | 100 | 15 |
|  | 1 | 15 | *O. cuniculus* | Chr 14 | 153,942,001 | 153,942,015 | 15 | 9.4 | 100 | 15 |
|  | 6 | 20 | *O. cuniculus* | Chr 3 | 31,637,091 | 31,637,105 | 15 | 9.4 | 100 | 15 |
|  | 6 | 20 | *O. cuniculus* | Chr 8 | 31,999,798 | 31,999,812 | 15 | 9.6 | 100 | 15 |
|  | 1 | 15 | *O. cuniculus* | Chr 4 | 102,798 | 102,812 | 15 | 9.7 | 100 | 15 |
|  | 6 | 20 | *O. cuniculus* | Chr 17 | 30,031,376 | 30,031,390 | 15 | 9.7 | 100 | 15 |
| 104 (Core PCR II) | 1 | 16 | *O. cuniculus* | Scaffold GL019385 | 21,194 | 21,209 | 16 | 1.3 | 100 | 16 |
|  | 1 | 15 | *O. cuniculus* | Chr 1 | 122,723,844 | 122,723,858 | 15 | 9.2 | 100 | 15 |
|  | 1 | 15 | *O. cuniculus* | Chr 2 | 60,608,826 | 60,608,840 | 15 | 9.3 | 100 | 15 |
|  | 2 | 16 | *O. cuniculus* | Chr 14 | 140,571,561 | 14,057,157 | 15 | 9.4 | 100 | 15 |
|  | 1 | 15 | *O. cuniculus* | Chr 13 | 253,507 | 253,521 | 15 | 9.4 | 100 | 15 |
| 134 (Core PCR II) | 3 | 20 | *O. cuniculus* | Chr 1 | 78,061,445 | 78,061,462 | 18 | 0,082 | 100 | 18 |
|  | 2 | 17 | *O. cuniculus* | Scaffold GL018761 | 1,198,578 | 1,198,593 | 16 | 1.3 | 100 | 16 |
|  | 5 | 20 | *O. cuniculus* | Scaffold GL018836 | 11,174 | 11,189 | 16 | 1.3 | 100 | 16 |
|  | 1 | 15 | *O. cuniculus* | Chr 2 | 159,378,698 | 159,378,712 | 15 | 9.3 | 100 | 15 |
|  | 4 | 18 | *O. cuniculus* | Chr 14 | 85,619,317 | 85,619,331 | 15 | 9.4 | 100 | 15 |
|  | 3 | 17 | *O. cuniculus* | Chr 14 | 58,133,961 | 58,133,975 | 15 | 9.4 | 100 | 15 |
|  | 4 | 18 | *O. cuniculus* | Chr 12 | 12,419,706 | 12,419,720 | 15 | 9.4 | 100 | 15 |
|  | 5 | 19 | *O. cuniculus* | Chr 10 | 7,778,574 | 7,778,588 | 15 | 9.9 | 100 | 15 |
| HVR1F (E1/E2) | --- | --- | *O. cuniculus* | --- | --- | --- | --- | --- | --- | --- |
|  | --- | --- | *M. musculus* | --- | --- | --- | --- | --- | --- | --- |
| HVR1R (E1/E2) | 1 | 17 | *O. cuniculus* | Chr 3 | 139,211,765 | 139,211,781 | 17 | 0.65 | 100 | 17 |
|  | 1 | 17 | *O. cuniculus* | Chr 12 | 134,023,078 | 134,023,094 | 17 | 0.65 | 100 | 17 |
|  | 1 | 17 | *O. cuniculus* | Chr 13 | 65,864,853 | 65,864,869 | 17 | 0.65 | 100 | 17 |
|  | 6 | 21 | *O. cuniculus* | Chr 3 | 40,956,398 | 40,956,413 | 16 | 3.7 | 100 | 16 |
|  | 6 | 21 | *O. cuniculus* | Chr3 | 35,742,367 | 35,742,382 | 16 | 3.7 | 100 | 16 |
|  | 6 | 21 | *O. cuniculus* | Chr3 | 34,986,831 | 34,986,846 | 16 | 3.7 | 100 | 16 |
|  | 7 | 22 | *O. cuniculus* | Chr 14 | 149,487,655 | 149,487,670 | 16 | 3.7 | 100 | 16 |
|  | 7 | 16 | *O. cuniculus* | Chr 9 | 72,091,120 | 72,091,135 | 16 | 3.8 | 100 | 16 |
|  | 4 | 19 | *O. cuniculus* | Chr 4 | 44,209,260 | 44,209,275 | 16 | 3.8 | 100 | 16 |
|  | 6 | 21 | *O. cuniculus* | Chr 11 | 23,950,609 | 23,950,624 | 16 | 3.8 | 100 | 16 |
|  | 5 | 20 | *O. cuniculus* | Chr 11 | 9,228,906 | 9,228,921 | 16 | 3.8 | 100 | 16 |
|  | 5 | 20 | *O. cuniculus* | Chr 20 | 6,807,876 | 6,807,891 | 16 | 3.8 | 100 | 16 |
|  | 6 | 21 | *O. cuniculus* | Scaffold GL018699 | 6,689,073 | 6,689,088 | 16 | 3.8 | 100 | 16 |
|  | 6 | 21 | *O. cuniculus* | Scaffold GL018719 | 760,921 | 760,936 | 16 | 3.8 | 100 | 16 |
|  | 6 | 21 | *O. cuniculus* | Scaffold GL018720 | 2,160,883 | 2,160,898 | 16 | 3.8 | 100 | 16 |
|  | 6 | 21 | *O. cuniculus* | Scaffold GL018731 | 906,300 | 906,315 | 16 | 3.8 | 100 | 16 |
| S (NS5A/B PCR I) | 14 | 30 | *O. cuniculus* | Chr 2 | 118,884,519 | 118,884,535 | 17 | 3.5 | 100 | 17 |
| AS (NS5A/B PCR I) | 5 | 24 | *O. cuniculus* | Scaffold GL018702 | 7,608,327 | 7,608,346 | 20 | 0.037 | 100 | 20 |
|  | 6 | 22 | *O. cuniculus* | Chr 11 | 7,608,346 | 107,101,489 | 17 | 3.2 | 100 | 17 |
| S` (NS5A/B PCR II) | 14 | 30 | *O. cuniculus* | Chr 2 | 118,884,519 | 118,884,535 | 17 | 3.5 | 100 | 17 |
| AS` (NS5A/B PCR II) | 12 | 29 | *O. cuniculus* | Chr 8 | 4,853,339 | 4,853,356 | 18 | 0.74 | 100 | 18 |
|  | 9 | 28 | *O. cuniculus* | Chr 1 | 90,930,394 | 90,930,414 | 17 | 3.2 | 95.24 | 21 |
|  | 4 | 20 | *O. cuniculus* | Chr 12 | 47,749,674 | 47,749,690 | 17 | 3.2 | 100 | 17 |
|  | 3 | 19 | *O. cuniculus* | Chr 11 | 71,955,734 | 71,955,750 | 17 | 3.2 | 100 | 17 |
|  | 9 | 29 | *O. cuniculus* | Scaffold GL018703 | 4,748,942 | 4,748,961 | 17 | 3.2 | 95.24 | 21 |
|  | 14 | 30 | *O. cuniculus* | Scaffold GL018850 | 490,978 | 490,994 | 17 | 3.2 | 100 | 17 |
| Pr3 (RdRp-NS5B) | --- | --- | *O. cuniculus* | --- | --- | --- | --- | --- | --- | --- |
| Pr4 (RdRp-NS5B) | --- | --- | *O. cuniculus* | --- | --- | --- | --- | --- | --- | --- |

Nucleotide homology between HCV specific primers used in PCRs assays and *O. cuniculus* genomic sequences deposited at the ensembl site (http://www.ensembl.org/Multi/blastview). Blastn was performed with selected DNA database and search sensitivity near - exact matches’ options. HCV specific primers, region and PCR assay: 256 (5' CGCGCGACTAGGAAGACTTC 3') and 186 (5' ATGTACCCCATGAGGTCGGC 3') for the core PCRI; 104 (5' AGGAAGACTTCCGAGCGGTC 3') and 134 (5' CCAAGAGGGACGGGAACCTC 3') for the core PCRII; HVR1F (5' TGCTGGGTCCARRTYACCCC 3') and HVR1R (5' GCTGTCATTACAGTTAAGGGCA 3') for the E1/E2 (HVR-1); S (5' TGGGGATCCCGTATGATACCCGCTGCTTTGA 3') and AS (5' GGCGGAATTCCTGGTCATAGCCTCCGTGAA 3') for the NS5A/B PCRI; S' (5' TGCGGTTATTGCCGTTGTCGCGCCAGCGG 3') and AS' (5' GGCAGAATACCTAGTCATGGCCTCTGTGAA 3') for the NS5A/B PCRII; Pr3 (5' TATGAYACCCGCTGYTTTGACTC 3') and Pr4 (5' GCNGARTAYCTVGTCATAGCCTC 3') for the RdRp-NS5B.
